# Application and prospects of organoid-on-a-chip in research on the intestinal mucosal barrier

**DOI:** 10.1093/burnst/tkag029

**Published:** 2026-04-09

**Authors:** Kun Qian, Yiwen Wang, Ran Li, Mengmeng Zhuang, Zhengnan Zhao, Chao Meng, Jiayun Zhou, Wensheng Wang, Yong Sun

**Affiliations:** Department of Burn Surgery, the Affiliated Huaihai Hospital of Xuzhou Medical University, No. 226 Tongshan Road, Yunlong District, Xuzhou, Jiangsu Province 221004, China; Department of Burn Surgery, The 71st Group Army Hospital of People’s Liberation Army, No. 226 Tongshan Road, Xuzhou, Jiangsu Province 221004, China; Department of Burn Surgery, the Affiliated Huaihai Hospital of Xuzhou Medical University, No. 226 Tongshan Road, Yunlong District, Xuzhou, Jiangsu Province 221004, China; Department of Burn Surgery, The 71st Group Army Hospital of People’s Liberation Army, No. 226 Tongshan Road, Xuzhou, Jiangsu Province 221004, China; Department of Burn Surgery, the Affiliated Huaihai Hospital of Xuzhou Medical University, No. 226 Tongshan Road, Yunlong District, Xuzhou, Jiangsu Province 221004, China; Department of Burn Surgery, The 71st Group Army Hospital of People’s Liberation Army, No. 226 Tongshan Road, Xuzhou, Jiangsu Province 221004, China; Department of Burn Surgery, the Affiliated Huaihai Hospital of Xuzhou Medical University, No. 226 Tongshan Road, Yunlong District, Xuzhou, Jiangsu Province 221004, China; Department of Burn Surgery, The 71st Group Army Hospital of People’s Liberation Army, No. 226 Tongshan Road, Xuzhou, Jiangsu Province 221004, China; Department of Burn Surgery, the Affiliated Huaihai Hospital of Xuzhou Medical University, No. 226 Tongshan Road, Yunlong District, Xuzhou, Jiangsu Province 221004, China; Department of Burn Surgery, The 71st Group Army Hospital of People’s Liberation Army, No. 226 Tongshan Road, Xuzhou, Jiangsu Province 221004, China; Department of Burn Surgery, the Affiliated Huaihai Hospital of Xuzhou Medical University, No. 226 Tongshan Road, Yunlong District, Xuzhou, Jiangsu Province 221004, China; Department of Burn Surgery, The 71st Group Army Hospital of People’s Liberation Army, No. 226 Tongshan Road, Xuzhou, Jiangsu Province 221004, China; Department of Burn Surgery, the Affiliated Huaihai Hospital of Xuzhou Medical University, No. 226 Tongshan Road, Yunlong District, Xuzhou, Jiangsu Province 221004, China; Department of Burn Surgery, The 71st Group Army Hospital of People’s Liberation Army, No. 226 Tongshan Road, Xuzhou, Jiangsu Province 221004, China; Department of Burn Surgery, the Affiliated Huaihai Hospital of Xuzhou Medical University, No. 226 Tongshan Road, Yunlong District, Xuzhou, Jiangsu Province 221004, China; Department of Burn Surgery, The 71st Group Army Hospital of People’s Liberation Army, No. 226 Tongshan Road, Xuzhou, Jiangsu Province 221004, China; Department of Burn Surgery, the Affiliated Huaihai Hospital of Xuzhou Medical University, No. 226 Tongshan Road, Yunlong District, Xuzhou, Jiangsu Province 221004, China; Department of Burn Surgery, The 71st Group Army Hospital of People’s Liberation Army, No. 226 Tongshan Road, Xuzhou, Jiangsu Province 221004, China

**Keywords:** Intestinal mucosal barrier, Intestinal organoids, Organ-on-a-chip, Organoid-on-a-chip, Gut–organ-axis-on-a-chip, Intestinal injury, Intestinal barrier repair

## Abstract

The integrity of the intestinal mucosal barrier is essential for maintaining normal gut physiology, and its disruption is associated with a wide range of conditions, including trauma- and burn-related intestinal injury, which remain difficult to manage clinically. Intestinal organoid-on-a-chip systems have emerged as advanced *in vitro* models that reproduce key features of the intestinal microenvironment and physiological function. These systems have shown promise for studying mucosal injury and repair, assessing therapeutic strategies, and supporting translational research. This review summarizes the basic principles of intestinal organoid-on-a-chip technology and examines its use in modeling intestinal barrier function, inflammatory responses, drug screening, regenerative approaches, and trauma-related barrier repair. It also reviews recent progress in preclinical studies, considers potential applications in gastrointestinal research, and discusses current technical challenges, particularly those related to scalability and reproducibility. Future directions for the development of next-generation systems are also outlined. With the continued integration of advances across disciplines, these platforms may provide useful tools for studying and treating disorders involving the intestinal mucosal barrier, especially in the context of trauma and burns.

## Highlights

- Provides a barrier-centered overview of intestinal organoid-on-a-chip technology and its major construction strategies.
- Summarizes how these platforms reconstruct epithelial, mucus, immune, microbial, and mechanical barrier functions.
- Highlights applications in mucosal injury and repair, as well as translational potential, current bottlenecks, and future directions.

## Background

The intestinal mucosal barrier is a dynamic structure that is composed of a mucus layer, epithelial cells, immune cells, the extracellular matrix (ECM), and intercellular junctional complexes, including tight junctions [1–4]. Its integrity is maintained by three main processes: continuous epithelial renewal driven by crypt stem cells; the regulation of permeability through intercellular junction proteins, such as claudins, occludin, and zonula occludens; and the control of epithelial proliferation and differentiation by the stem cell niche, including Paneth cells, stromal cells, and multiple growth factors [1–4]. This barrier is central to normal gut function. It limits the entry of harmful luminal contents into host tissues while allowing nutrient absorption, preserving microbiota homeostasis, and regulating mucosal immunity [5–9]. When impaired, it may contribute to intestinal inflammation, metabolic disorders, and systemic disease, which makes barrier injury and repair important areas of study [8–12].

Traditional *in vitro* models have contributed substantially to research on intestinal mucosal barrier injury, but important limitations remain. Animal models provide system-level information; however, species differences and ethical concerns limit their applicability, and they do not fully reproduce the physiological and pathological features of the human intestine [13]. Commonly used immortalized intestinal epithelial cell (IECs) lines, such as Caco-2 and HT-29, are convenient to use [14–15], but they lack cellular diversity, apical basal polarity, and physiological mechanical cues [16]. In addition, two-dimensional static culture systems cannot reproduce the three-dimensional (3D) crypt-villus structure or its dynamic interactions with the immune system and microbiota [17]. These limitations support the need for more advanced *in vitro* models of the intestinal mucosal barrier.

Organoid culture and organ-on-a-chip (OoC) technologies have expanded the available approaches for modeling the intestinal barrier. Intestinal organoids derived from tissue stem cells or induced pluripotent stem cells (iPSCs) self-organize into miniature intestinal structures. They contain multiple cell types and retain important functional features of native intestinal tissue, allowing them to recapitulate key aspects of intestinal structure and physiology [18–19]. OoC systems are biomimetic platforms built on microfluidic devices. By controlling the 3D organization of living cells and their surrounding microenvironment, these systems can reproduce major architectural and physiological features of human organs [18, 20]. Intestinal organoid-on-a-chip technology combines these two approaches on microfluidic platforms to generate more complex and physiologically relevant intestinal models [18]. This approach has advanced the development of intestinal disease models and supported studies of mucosal injury mechanisms, drug development, and precision medicine [18, 21].

In this review, we summarize recent advances in intestinal organoid-on-a-chip technology, with a particular focus on its use in the study of intestinal mucosal barrier injury. We first outline the structural and functional features of the intestinal mucosal barrier and its roles in health and disease. We then review the current fabrication strategies for intestinal organoid-on-a-chip platforms and recent progress in the reproduction of key barrier functions. We also discuss the broader applications of this technology, including disease modeling, especially models of intestinal mucosal injury, drug screening, regenerative medicine, and multi-organ interactions. Finally, we consider the challenges and future directions of this field and discuss next-generation intestinal organoid-on-a-chip models that are informed by advances in biology, engineering, and materials science.

## Review

### The intestinal mucosal barrier: structure, disease relevance, and modeling challenges

The intestinal mucosal barrier is a multilayered, dynamically regulated system that separates luminal contents from underlying tissues and helps maintain homeostasis. It is not a single structural unit but a coordinated system composed of mechanical, chemical, immune, and microbial components that preserve selective permeability, limit pathogen invasion, and support host-microenvironment interactions [7, 22–24]. Barrier dysfunction is associated with a wide range of intestinal and systemic diseases, including inflammatory bowel disease (IBD) [10], irritable bowel syndrome (IBS) [25], necrotizing enterocolitis [26], metabolic disorders such as diabetes [11] and obesity [27], and cardiovascular disease [12]. In severe trauma and burn injury, loss of barrier integrity may further worsen systemic inflammation and contribute to secondary organ dysfunction [8–9, 28–29].

The mechanical barrier is formed mainly by IECs and their intercellular junctions, including tight junctions, adherens junctions, desmosomes, and gap junctions [30–31]. Tight junctions are the main regulators of paracellular permeability and consist of transmembrane proteins and cytoplasmic scaffold proteins that control the passage of water, ions, and macromolecules between adjacent cells [32–33]. In trauma and burn injury, oxidative stress, ischemia–reperfusion, and excessive inflammatory signaling can disrupt tight junction organization, increase epithelial permeability, and promote the development of a leaky gut [6, 34–37]. These changes are central to barrier failure but are difficult to reproduce in conventional static models.

The chemical barrier consists primarily of the mucus layer that covers the intestinal epithelium, together with mucins and other secreted protective factors [7, 30–31]. This gel-like layer prevents direct contact between luminal microbes and IECs, while coordinated mucus turnover and intestinal motility promote microbial clearance [7]. Mucins secreted mainly by goblet cells contribute to both physical separation and the regulation of microbial behavior. Antimicrobial peptides released by Paneth cells accumulate within the mucus layer and further strengthen local defense [31, 38]. In critical illness, including trauma and burns, mucus depletion, goblet cell dysfunction, and altered secretion of protective mediators may weaken this first line of luminal defense [7, 28, 30–31, 39]. These injury-related secretory changes are also not well reproduced by reductionist *in vitro* systems.

The immune barrier is a specialized local defense system composed of IECs, gut-associated lymphoid tissue (GALT), lamina propria lymphocytes, and effector molecules such as secretory immunoglobulin A (sIgA) [31, 40]. Secretory IgA neutralizes pathogens and limits epithelial adhesion without causing excessive inflammation, whereas intraepithelial lymphocytes and regulatory T cells help maintain immune surveillance and tolerance to commensal bacteria and food antigens [7, 31]. Closely related to this, the biological barrier consists of the symbiotic gut microbiota and its metabolites. These compounds protect against pathogen colonization by competing for nutrients and ecological niches and by producing metabolites such as short-chain fatty acids (SCFAs) that support epithelial metabolism and barrier integrity [41–44]. Dysbiosis disrupts the coordination between microbial, physical, and immune defenses and thereby increases the susceptibility to infection and inflammation [45]. In trauma- and burn-associated injury, excessive inflammatory activation, cytokine imbalance, bacterial translocation, and related microbial disturbances may together worsen barrier dysfunction and systemic inflammatory response syndrome (SIRS) [5–9, 29, 34, 39, 46].

Once barrier integrity is lost, increased epithelial permeability allows bacteria, toxins, and undigested antigens to cross the epithelium, triggering immune responses, and amplifying systemic inflammation [5–9]. In severe trauma and burn injury, this process may be further intensify, linking barrier failure to bacterial translocation, SIRS, and secondary organ dysfunction [8–9, 28–29, 46].

Taken together, these findings suggest that the intestinal mucosal barrier is an integrated, dynamically regulated system in which dysfunction reflects coordinated disturbances in epithelial integrity, mucus protection, immune regulation, and host-microbiota interactions. This complexity is particularly relevant in trauma and burn settings, where barrier disruption is dynamic and involves multicellular and biomechanical responses [28–29, 35–36, 47]. It also helps explain why traditional two-dimensional epithelial cultures, static Transwell (TW) systems, and conventional tissue models often fail to reproduce the structural organization and cellular interactions involved in barrier injury and repair. More physiologically relevant and controllable *in vitro* platforms are therefore needed to study these processes, which provides a strong rationale for the development of intestinal organoid-on-a-chip systems.

### Integration of organoid culture and OoC technologies

**Figure 1:**
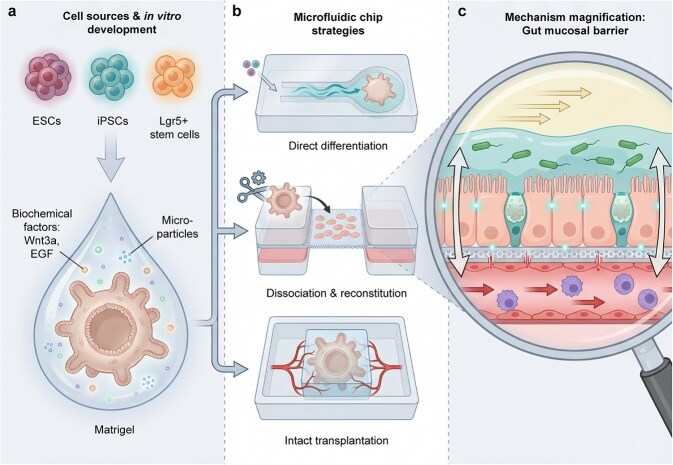
Conceptual framework for intestinal organoid-on-a-chip systems: cell sources, engineering strategies, and barrier-oriented design. (**a**) Cell sources and *in vitro* organoid development from ESCs, iPSCs, and ASCs. (**b**) Major chip-construction strategies, including direct on-chip differentiation, dissociation and reconstitution, and intact organoid transplantation. (**c**) Barrier–oriented design features for reconstructing the intestinal mucosal barrier, including epithelial organization, luminal flow, microbial exposure, and immune-associated interactions. ASCs can be directly seeded onto the chip. *ESCs* embryonic stem cells, *iPSCs* induced pluripotent stem cells, *ASCs* adult stem cells

In this context, intestinal organoid-on-a-chip systems are being developed to preserve intestinal tissue specificity while allowing precise control of the microenvironment (Figure 1).

### Organoid technology

Organoid technology is a core approach for generating 3D miniature organ models *in vitro* through the self-organization of stem cells [48–51]. It relies on stem cells, including multipotent adult stem cells such as intestinal Lgr5+ stem cells, embryonic stem cells (ESCs), and iPSCs, all of which can give rise to intestinal organoids under defined biochemical conditions and matrix support (Figure 1a) [50]. Under directed differentiation conditions, these cells form polarized organoid structures that contain multiple functional cell types, including absorptive and goblet cells, and reproduce key features of the crypt-villus unit [52–53]. Successful organoid culture depends on both an appropriate 3D matrix scaffold and defined growth factor conditions. Matrigel remains widely used as a scaffold, although its application is limited by batch-to-batch variability, animal origin, and poorly defined composition [54–56]. Synthetic hydrogels, including functionalized polyethylene glycol (PEG) and polyisocyanide-invasin hydrogels (PIC-INV), have therefore been developed to support efficient organoid formation and long-term expansion [57–61]. The classic ENR growth factor cocktail, composed of epidermal growth factor (EGF), Noggin, and R-spondin, supports stem cell self-renewal and differentiation, whereas bone morphogenetic protein (BMP) inhibitors, Wnt agonists, and related molecules help regulate lineage-specific development [62–65]. Organoids are now widely used in disease modeling, drug screening, toxicology, and studies of host-microbiome interactions [65–68].

For intestinal applications, however, conventional organoid systems still have important limitations. Intestinal organoids usually form closed 3D structures with the lumen facing inward, which limits direct access to the apical surface and complicates studies of nutrient exposure, microbial colonization, drug absorption, and mucus dynamics. Standard static culture cannot reproduce luminal flow, vascular perfusion, or peristalsis-like mechanical stimulation, which are important for epithelial maturation, barrier maintenance, and normal intestinal function. Long-term culture is also constrained by limited nutrient and oxygen diffusion. In addition, stromal, immune, endothelial, and microbial components remain incompletely represented in most conventional organoid systems [18, 20, 69–71].

### OoC technology

OoC technology is an emerging microfluidic platform for cell culture. By combining microengineering with cell biology, it can reproduce key aspects of human organ structure and the microenvironment at the microscale and thereby model physiological and pathological processes under controlled conditions [72–73]. Compared with traditional two-dimensional culture, it supports dynamic cell–cell and cell-environment interactions and reduces some of the limitations of animal models, including interspecies differences and ethical concerns [72–74]. OoC systems typically integrate microfluidics, biomaterials, and multiple cell sources. Microfluidic channels can mimic blood flow and shear stress, influence cell alignment and function, improve nutrient delivery and waste removal, and support real-time monitoring [75–81]. Biomaterial selection requires a balance between biological relevance and functional performance. Polydimethylsiloxane (PDMS) is widely used because of its optical transparency and biocompatibility, although surface modification is often needed to reduce nonspecific molecular adsorption [82–84]. Natural hydrogels such as collagen and gelatin methacryloyl (GelMA) can mimic ECM cues and support cell adhesion and differentiation, but their limited mechanical strength often requires combination with synthetic polymers [85–86]. The choice of cell source also affects physiological relevance. Primary cells retain mature functions but are limited in availability, whereas iPSCs provide a renewable source of human cells through directed differentiation and enable patient-specific disease modeling [87–90].

In intestinal models, the main advantage of OoC systems is that they provide capabilities that are not available in conventional organoid culture. These include controlled luminal and vascular flow; apical-basal compartmentalization; defined oxygen and nutrient gradients; cyclic mechanical deformation resembling peristalsis; and the incorporation of endothelial, immune, stromal, or microbial components in an organized spatial arrangement [69–71, 91–93]. This makes chip-based systems useful for studying flow-dependent epithelial maturation, barrier polarization, host–microbe interactions, and mechanically regulated intestinal physiology.

### Organoid-on-a-chip technology

**Figure 2:**
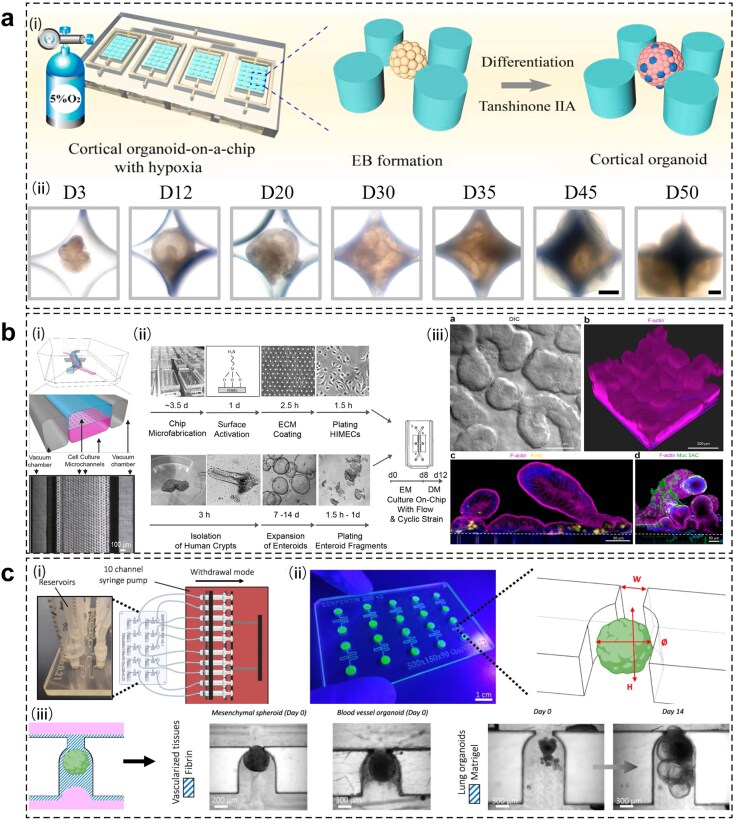
Representative construction strategies for organoid-on-a-chip platforms. (**a**) On-chip direct differentiation of hiPSCs into cortical organoids under hypoxic conditions. Adapted with permission [97]. Copyright 2023, Science and Technology Review Publishing House. (**b**) Reassembly on chip after intestinal organoid dissociation, with enteroid fragments seeded and co-cultured with microvascular endothelial cells to form an epithelial barrier. Adapted with permission [91]. Copyright 2018, Springer Nature. (**c**) Whole-organoid trapping and culture in a parallel microfluidic platform. Adapted with permission [79]. Copyright 2024, Springer Nature. *EB* embryoid body, *HIMECs* human intestinal microvascular endothelial cells, *EM* Eagle’s medium, *DM* Dulbecco’s modified Eagle’s medium, *hiPSCs* human induced pluripotent stem cells, *ECM* extracellular matrix

Organoids alone and OoC systems alone capture only part of human organ complexity and systemic interactions. Seeding primary cells or organoid-derived cells onto chip platforms provides a more integrated approach [69]. In some intestinal models, adult stem cells (ASCs) or ASC-derived epithelial cells can also be directly seeded onto chip platforms, depending on the experimental design (Figure 1a and b). Organoid-on-a-chip technology combines the advantages of both systems. Early work by Clevers and colleagues on gastrointestinal organoids laid important groundwork for later studies of barrier tissues, including the intestine [18], lung [94], and brain [95]. Current construction strategies include direct on-chip differentiation, reassembly after organoid dissociation, and the transplantation of intact organoids, with the latter two being used more commonly (Figures 1b and 2).

For intestinal applications, this integration is particularly useful because it helps address key limitations of conventional organoid culture, including restricted luminal access, limited mechanical stimulation, inadequate vascular support, and incomplete multicellular complexity, thereby improving the physiological relevance of intestinal barrier models [69–71, 91].

#### On-chip direct differentiation

Direct on-chip differentiation involves seeding pluripotent stem cells, such as iPSCs or ESCs, directly into OoC platforms (Figure 1b). Campisi *et al.* developed a 3D blood–brain barrier model using self-organized human iPSC-derived endothelial cells together with primary pericytes and astrocytes and used this system to study barrier structure and molecular transport [96]. Zhi *et al.* generated cortical organoids on microcolumn array chips to model fetal brain development and reported that tanshinone IIA promoted neural differentiation and functional network maturation (Figure 2a) [97]. This strategy allows stem cell differentiation and self-organization to occur directly within a biomimetic microenvironment and is therefore useful for studying developmental processes and microenvironment-dependent patterning. Its capacity to generate structurally mature and highly complex tissues may, however, be limited, and some degree of pre-differentiation is often still required [96–97]. In intestinal research, this approach is conceptually attractive for studying epithelial development and microenvironment-guided lineage specification, but practical applications remain less common than with the other two strategies, partly because intestinal differentiation requires tight control of niche factors, epithelial polarity, and regional patterning.

#### Reassembly on a chip after organoid dissociation

This strategy involves dissociating mature organoids into single cells or small clusters before they are seeded onto chip platforms (Figure 1b) [91, 98]. Its main advantage is the greater control it provides over the placement of organoid-derived cells, epithelial organization, and barrier formation. The dissociation process, however, can damage cells and disrupt cell–cell junctions, with partial loss of native organoid architecture [91, 98]. Kasendra *et al.* enzymatically dissociated human intestinal organoids into single cells and fragments and then seeded them onto chips to generate a villus-like intestinal epithelial model. They reported that compared with single cells, organoid fragments resulted in higher epithelial formation rates and more efficient continuous barrier formation than single cells (Figure 2b) [91]. Ma *et al.* used mechanically disrupted and magnetically sorted LTL+ kidney organoid cells to establish a polarized tubular epithelial monolayer on a chip that reproduced key physiological features of the kidney [98]. In intestinal systems, this strategy improves access to the apical epithelial surface and supports continuous epithelial barrier formation for permeability testing, microbial stimulation, drug transport studies, and TEER-based assessment.

#### Whole organoid transplantation on chip

The transplantation of intact organoids is another major strategy for constructing organoid-on-a-chip systems (Figure 1b). In static encapsulation approaches, organoids are embedded in hydrogels or ECM before transplantation, which helps reduce tissue damage. The optimization of matrix mechanics and nutrient diffusion can support culture for more than 2 weeks and may be compatible with high-throughput applications [70, 99–100]. This strategy better preserves the native 3D architecture and multicellular composition of organoids but is limited by insufficient nutrient and oxygen diffusion, which can lead to central necrosis and reduced long-term viability, particularly under static conditions [70, 99–100]. Dynamic perfusion platforms have been developed to address this problem. Quintard *et al.* achieved deep perfusion of organoids by promoting anastomosis between human umbilical vein endothelial cell (HUVEC) vascular beds and capillaries from human vascular organoids, allowing them to be cultured for more than 30 days (Figure 2c) [79]. Josep *et al.* used a similar strategy to establish a 3D neurovascular model that reduced core necrosis in brain organoids and extended viability beyond 40 days [101]. These vascularization strategies improve both survival and physiological relevance [102–103]. In intestinal models, intact organoid transplantation is particularly useful when the preservation of crypt-villus-like spatial organization, multicellular composition, or higher-order tissue architecture is the main priority. Its dependence on diffusion for oxygen and nutrient delivery also highlights the importance of perfusion support and vascularization in this setting.

#### Comparison of construction strategies

Each of the three construction strategies has distinct advantages and limitations. On-chip direct differentiation is well suited to studies of development and microenvironment-dependent patterning, but it may be less effective for generating structurally mature and highly complex tissues. Reassembly after organoid dissociation provides better control over cell seeding, epithelial organization, and barrier formation, but dissociation can damage cells and partially disrupt the native architecture. Whole organoid transplantation more effectively preserves 3D structure and multicellular composition but is more vulnerable to limited oxygen and nutrient diffusion, especially under static encapsulation, which may reduce long-term viability [70, 91, 96–103]. Dynamic perfusion and vascularization can partly address these limitations and improve physiological relevance, although at the cost of greater technical complexity [79, 101–103]. Accordingly, the choice among these strategies should be guided by the experimental objective, whether the aim is developmental modeling, epithelial barrier reconstruction, or preservation of higher-order organoid complexity. Intestinal research also depends on which limitations of conventional organoids are being addressed, such as apical access and barrier analysis, physiologically relevant flow and mechanical stimulation, or preservation of the native 3D epithelial architecture. The key question is whether these systems can faithfully reproduce the coordinated functions of the intestinal mucosal barrier.

### Simulation of intestinal barrier functions by organoid-on-a-chip technology

Intestinal organoid-on-a-chip technology has become an increasingly advanced platform for reproducing major barrier functions and the biomechanical microenvironment of the human intestine (Table 1) [92–93, 104–115]. As shown in Figure 1c, these systems are designed to reconstruct epithelial organization together with luminal flow, microbial exposure, and immune-related interactions that are difficult to capture in conventional *in vitro* models. Current studies focus on the mechanical, chemical, immune, and biological barriers, as well as the physical cues that regulate epithelial organization, barrier integrity, and host-microenvironment interactions. Because these components function as an integrated system, the discussion below moves from epithelial structure to luminal defense, then to immune and microbiota-related regulation, and finally to the mechanical cues that coordinate these processes.

**Table 1:** Simulation of the four intestinal barrier functions and mechanical stimulation of the intestinal chip

Chip types	Cell types	Device material	Manufacturing method	Research content	Main findings	References
Double-layered microfluidic chip	Caco-2 cells, U937 cells	PDMS, PS, PMMA, PET	Microfluidic technology, dynamic culture system	Caco-2/U937 cells coculture, IECs, Intestinal mucosal barrier permeability	Designed a microfluidic -based dynamic *in vitro* model of the human intestinal barrier.	[104]
Membrane-on-chip	Caco-2 cells	PDMS, PMMA	Microfluidic technology	ECs , Intestinal villi	Designed an In-Crypts microfluidic system and reconstructed the microscale villous organization and functionality of the small intestine.	[105]
CNF-3D intestinal chip	Caco-2 cells	PDMS	Microfluidic technology, dynamic culture system, 3D printing technology	IECs, Intestinal villi, Intestinal mucosal barrier permeability	Designed an array of carbon nanofiber bundle-based 3D *in vitro* intestinal microvilli.	[106]
HFM gut-on-a-chip	Caco-2 cells	PET	Microfluidic technology, ALI-VIP, LLI-NT, LLI-VIP, ALI-NT	IECs, Intestinal mucosal barrier permeability Induce directed differentiation and mucus secretion	Designed a model *in vitro* intestinal, intestinal mucous membrane barrier function and intestinal microbial interactions.	[107]
Primary human intestine chip	HCoEpC	PDMS	Microfluidic technology, TW culture inserts	Induce goblet cell differentiation and mucus secretion	Colonic mucus with physiologically relevant bilayer structures is generated *in vitro.*	[108]
Hydrogel-integrated millifluidic system	HT29-MTX cells, ISCs	dSIS-MA biomaterial resin, Hydrogel	Microfluidic technology (hydrogel-integrated), static/dynamic culture system, 3D printing technology	Multi-lineage directed differentiation of ISCs	The combination of dSIS-MA-Based hydrogel scaffolds and physiological shear stress can promote multi-lineage differentiation.	[109]
Emulate duodenum-chip	Human duodenal epithelial cells, HIMECs	PDMS	Microfluidic technology, dynamic culture system,	Intestinal mucosal barrier	The down-regulation of IFI6 after MNPs' absorption leads to impaired immune function.	[110]
Intestine-on-chip	Caco-2 cells, HUVECs, PBMCs	-	Microfluidic technology, dynamic culture system,	Intestinal mucosal barrier, Co-culture of immune cells	Co-culture models are an efficient platform for studying the responses of the intestinal immune system to bacterial and fungal colonization and infection.	[111]
3D Perfused intestine-on-a-chip	Caco-2 cells HT29-MTX cells, THP-1 cells, MUTZ-3 cells	-	Microfluidic technology(Gravity-driven OrganoPlate three-lane), dynamic culture system	Intestinal inflammation, Intestinal mucosal barrier	Treatment of cultures with the anti-inflammatory compound TPCA-1 can prevent inflammatory states in cells.	[112]
Leaky gut chip	Caco-2 cells BBEs	PDMS	Microfluidic technology, dynamic culture system, mechanical stimulation	Probiotic co-culture, Intestinal mucosal barrier	Designed a chip intestinal leakage model, validation of probiotic intestinal barrier repair and anti-inflammatory effects.	[113]
A microfluidic co-culture model	HCT116 cells, THP-1 cells	PDMS	Microfluidic technology 3D printing technology	Co-culture of multicellular and complex microbial communities	Inulin enhances the decline in colon cell survival mediated by macrophages in a microbiota-dependent manner.	[114]
Fully 3D-printed insert culture chamber	HT29-MTX cells	Resin	Static/Dynamic culture system, 3D printing technology	Goblet cell differentiation and mucus production, Fluid shear force	3D villous structures with highly proliferating apically under dynamic culture conditions. The thickness of the mucus layer increases under flow.	[92]
Human stomach-on-a-chip	hPSCs	PDMS	Microfluidic technology, dynamic culture system, 3D printing technology	Peristaltic motion	Designed an innovative, low cost, microfluidic platform, integrated with *in vitro* crawling stomach-on-a-chip.	[93]
Emulate intestine-chip	Caco-2 cells	PDMS	Microfluidic technology, dynamic culture system,	Co-culture of Caco-2 cells and Shigella Peristaltic motion	Shigella efficiently invades the intestinal tract by taking advantage of the microstructure and mechanical forces of the intestinal microenvironment.	[115]

*PS* polystyrene, *PMMA* polymethyl methacrylate, *PET* polyethylene terephthalate, *CNF* carbon nanofibers, *HFM* hollow fiber membrane, *LLI* liquid–liquid interface, *NT* no treatment, *HCoEpC* human colonic epithelial cells, *MTX* methotrexate, *ISCs* intestinal stem cells, *dSIS-MA* decellularized small intestinal submucosa-methacrylamide, *IFI6* interferon-inducible protein 6, *MNPs* micro-nanoplastics, *PBMCs* peripheral blood mononuclear cells, *THP-1* human monocytic leukemia cell line 1, *MUTZ-3* human acute myeloid leukemia cell line 3, *TPCA-1* 2-[(aminocarbonyl)amino]-5 -(4-fluorophenyl)-3- thiophenecarboxamide, *BBEs* brush border expressing cells, *HCT116* human colorectal cancer cell line 116, *hPSCs* human pluripotent stem cells

### Simulation of the mechanical barrier

The complex architecture of the intestine and the high aspect ratio of villi make *in vitro* reconstruction of intestinal microanatomy challenging [31, 69, 116]. Early intestinal chip models mainly supported epithelial monolayers in culture chambers or on basement membrane-like substrates and had limited structural and cellular complexity [104]. To address this, several 3D microvillus-like scaffolds have been developed [117–118], although many still do not achieve physiologically relevant cellular compartmentalization [69]. Wang *et al.* induced crypt-villus polarity and cellular compartmentalization using micropatterned crosslinked collagen scaffolds [119]. Another group generated villus-like hydrogel scaffolds by photolithography and observed spatially distinct differentiation along the villus axis [120]. Newer microfluidic approaches, including In-Crypts and carbon nanofiber bundle arrays (CNF-BAs), have further enabled the reconstruction of microscale villus architecture and function in the small intestine [105–106]. When combined with dynamic perfusion that mimics shear stress and villus motion, these systems further improve the simulation of the mechanical barrier [106, 109].

Different chip designs involve clear trade-offs. Simpler monolayer-based platforms offer better reproducibility and easier functional readouts, such as permeability and TEER, but do not reproduce crypt-villus organization or regional differentiation well. In contrast, scaffold-assisted and microengineered villus systems capture spatial architecture and epithelial compartmentalization more effectively, but are more difficult to standardize and may still not fully reproduce the multicellular composition of the native intestine. No current platform fully combines architectural realism, cellular diversity, and experimental robustness. Effective barrier modeling also requires the reconstruction of luminal defense functions, particularly those mediated by the mucus layer and epithelial secretory activity.

### Simulation of the chemical barrier

Reconstruction of the chemical barrier is another major goal of intestinal organoid-on-a-chip systems. Beyond epithelial morphology, these platforms aim to model key luminal defense functions, including goblet cell-derived mucus secretion, antimicrobial peptide production, and epithelial secretory responses under controlled microphysiological conditions [31, 38, 105, 109, 121]. This is important because the mucus layer and related secretory factors are dynamic and are influenced by inflammatory, microbial, and biomechanical cues [31, 38, 105, 109, 121], which are difficult to reproduce in conventional static models [69, 105, 109]. Several *in vitro* platforms have therefore been developed to better model these functions [105, 109]. Cecilia *et al.* optimized a microarray-based 3D crypt model to enhance goblet cell differentiation, thereby generating a double-layered mucus structure similar to that seen *in vivo*. Floor *et al.* established an intestinal barrier model using Caco-2 cells and reported that culture at the air–liquid interface (ALI) with vasoactive intestinal peptide (VIP) culture promoted goblet cell differentiation and mucus secretion [107]. These studies support the use of organoid-on-a-chip systems to investigate the disruption and restoration of chemical barrier function under pathological conditions.

Mucus reconstruction, however, remains inconsistent across platforms. Some systems are more effective at promoting goblet cell differentiation and mucus accumulation, whereas others are better suited for studies of epithelial transport or microbial exposure. In many current models, the biochemical composition, turnover dynamics, and regional heterogeneity of mucus are still not fully reproduced. Simulation of the chemical barrier should therefore be judged not only by the presence of mucus but also by how well a platform reproduces mucus organization, secretory dynamics, and interactions with epithelial and microbial factors. Even so, epithelial secretion alone cannot fully reproduce mucosal defense, which also depends on immune surveillance and inflammatory regulation.

### Simulation of the immune barrier

The stable long-term integration of immune cells into intestinal organoid-on-a-chip systems remains technically challenging. Co-culture of IECs with vascular endothelial cells provides an important basis for the subsequent incorporation of immune components [91]. On this basis, multiple models related to IBD have been developed to mimic disruption of the intestinal immune barrier [53, 110, 122–125]. Systems that also incorporate the gut microbiota provide a broader framework for studying immune barrier regulation and inflammatory crosstalk.

A major limitation is that many immune-integrated systems capture inflammatory activation more readily than long-term immune homeostasis or tolerance. They are often more useful as injury-response models than as complete models of physiological mucosal immune regulation. In addition, studies vary substantially in terms of immune cell source, co-culture duration, and endpoint selection, which limits direct comparison and standardization. Future progress will depend not only on incorporating more immune components, but also on establishing more consistent design principles and functional benchmarks. These challenges become greater when host-microbiota interactions are incorporated into barrier models.

### Simulation of the biological barrier

Reconstruction of host-microbiota interactions remains among the most important and technically demanding aspects of modeling the intestinal biological barrier [41–45]. In the native intestine, the commensal microbiota interacts dynamically with epithelial cells, mucus, immune mediators, and luminal metabolites to maintain barrier homeostasis [41–45]. These interactions are highly sensitive to oxygen gradients, nutrient supplies, flow conditions, and microbial compositions, making reproduction difficult in conventional static systems [69, 126–127]. Intestinal organoid-on-a-chip platforms therefore provide a more physiologically relevant approach for investigating how microbiota-associated signals contribute to barrier maintenance, dysbiosis, and disease progression. Continuous perfusion can establish nutrient and oxygen gradients that resemble those of the intestinal microenvironment *in vivo* [69, 113] while also supporting nutrient delivery, waste removal, and shear stress which promote epithelial differentiation and maturation [128]. They also reproduce microbial colonization and migration more effectively than traditional models do, providing useful insight into microbiota–host crosstalk [103, 113–114, 129–130].

Even so, simulation of the biological barrier remains limited by several unresolved challenges. The stable long-term co-culture of human epithelium with complex microbial communities remains difficult, particularly when strict anaerobic conditions are required. In addition, many current platforms use simplified microbial consortia rather than the broader ecological complexity of the human gut microbiome. As a result, existing studies are useful for mechanistic investigations but still reproduce only part of the ecological and metabolic complexity of host-microbiota interactions *in vivo*. In addition to microbial and cellular interactions, barrier function is also strongly shaped by the intestinal mechanical microenvironment.

### Simulation of the mechanical microenvironment

In addition to epithelial, immune, and microbiota-related factors, intestinal barrier function is closely regulated by the mechanical microenvironment *in vivo* [128, 131–132]. Fluid shear stress promotes cell differentiation and polarity, maintains barrier function, and regulates mucus secretion [92–93, 133]. Cyclic mechanical stretching also contributes to villus formation and activates the YAP/TAZ pathway to regulate stem cell proliferation and differentiation [134–135]. Organoid self-organization depends on internal tension and external tensile forces, which regulate stem cell renewal and differentiation through the PIEZO channel [136–138]. Intestinal organoid-on-a-chip platforms therefore offer clear advantages in reproducing the mechanical microenvironment underlying these processes [71–72, 75]. The modulation of matrix stiffness, viscoelasticity, and other physical properties within microfluidic systems can further influence cellular behavior [58, 139].

Different studies use markedly different flow rates, stretching modes, matrix properties, and mechanical readouts, making it difficult to determine which conditions are most physiologically relevant for specific intestinal applications. Thus, despite substantial progress, a major limitation remains the lack of unified standards that link chip-imposed mechanical cues to defined intestinal phenotypes and functional outcomes. These reconstructed functions are important not only for engineering design but also because they provide the basis for disease modeling, therapeutic evaluation, and translational application.

## Applications and validation of organoid-on-a-chip technology in intestinal mucosal barrier dysfunction

**Figure 3:**
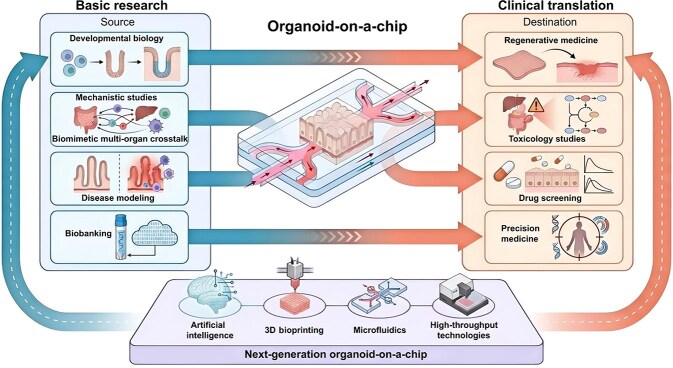
Translational pathway and next-generation development of intestinal organoid-on-a-chip. Intestinal organoid-on-a-chip serves as a bridging platform linking basic research to clinical translation. In basic research, it supports developmental biology, mechanistic studies, disease modeling, and biobanking; in clinical translation, it enables regenerative medicine, toxicology studies, drug screening, and precision medicine. AI, 3D bioprinting, microfluidics, and high-throughput technologies provide the technical foundation for next-generation platforms

Building on the barrier-reconstructive features described above, organoid-on-a-chip technology has become an advanced *in vitro* platform for studying intestinal mucosal barrier dysfunction because it combines the tissue specificity and self-organizing capacity of organoids with the controllability of microfluidic systems [140–141]. Compared with conventional two-dimensional cultures, TW systems, and static tissue engineering models, this technology more effectively reproduces key features of the intestinal microenvironment, including luminal flow, epithelial polarity, multicellular interactions, and biochemical gradients, thereby providing a more physiologically relevant framework for investigating barrier injury, repair, and therapeutic responses (Figure 3; Table 2).

**Table 2:** Comparison between organoid-on-a-chip technology and conventional tissue-engineering approaches in studies of intestinal mucosal barrier dysfunction

Dimension	Conventional tissue-engineering approaches	Organoid-on-a-chip technology	Relevance to intestinal mucosal barrier dysfunction research
Model basis	Commonly based on 2D cultures, TW systems, or scaffold-assisted 3D models	Integrates organoid self-organization with microfluidic engineering control	Better preserves intestinal epithelial specificity while enabling environmental regulation
Culture mode	Predominantly static culture	Supports continuous perfusion and dynamic culture	More closely mimics luminal flow and basolateral exchange *in vivo*
Mechanical cues	Usually absent or poorly controlled	Allows controllable shear stress and other biomechanical stimulation	Facilitates epithelial polarity, differentiation, and tight junction maintenance
Cellular complexity and interaction	Limited cell types and simplified intercellular communication	Supports co-culture with epithelial, endothelial, immune, and stromal cells	More suitable for reconstructing multicellular interactions involved in barrier homeostasis and injury
Microenvironmental recapitulation	Limited simulation of flow, gradients, matrix signals, and tissue interfaces	Enables integration of perfusion, mechanical cues, extracellular matrix, and biochemical gradients	More closely resembles the intestinal microphysiological environment
Barrier architecture reconstruction	Can form a basic epithelial layer, but polarity and higher-order structures are limited	Better reproduces epithelial polarity, apical-basal compartmentalization, and barrier interfaces	More suitable for studying barrier disruption and repair
Barrier function assessment	Mainly relies on endpoint assays, such as staining or single-time-point permeability tests	Allows dynamic assessment using transepithelial electrical resistance, tracer permeability, and live imaging	Enables continuous and multidimensional functional evaluation
Inflammation and infection modeling	Can reproduce selected pathological features but with limited complexity	Better suited for modeling cytokine exposure, pathogen challenge, and host–microbe interactions	More informative for investigating mechanisms of barrier dysfunction
Mechanistic resolution	Often limited to phenotypic observation and endpoint comparison	Enables dynamic readouts of temporal changes and causal relationships during barrier injury	Better suited for dissecting the coupling between inflammation, metabolic disturbance, and barrier breakdown
Drug screening and toxicological evaluation	Useful for preliminary efficacy and toxicity screening	More suitable for evaluating anti-inflammatory, antioxidant, barrier-restorative drugs, as well as functional toxicity	Improves physiological relevance in preclinical candidate evaluation
Translational potential	Widely used in basic research, but predictive power for clinical response is limited	Can be integrated with gut–liver axis models, multi-organ platforms, and patient-derived organoids	Offers greater value for translational research and precision medicine
Standardization and reproducibility	Protocols are relatively mature and generally show better cross-study consistency	More susceptible to batch-to-batch variation and inter-platform variability due to combined biological and engineering heterogeneity	Standardization is essential for reliable mechanistic interpretation, cross-study comparability, and translational adoption

### Disease modeling and mechanistic investigation

Organoid-on-a-chip technology has become an important tool for modeling epithelial barrier injury across inflammatory, infectious, metabolic, and systemic injury-related conditions [21, 35–36, 47, 69, 141–148]. Unlike static culture systems, these platforms allow controlled perfusion and biomechanical stimulation, both of which are important for maintaining epithelial differentiation, polarity, and tight junction stability. This dynamic control allows a more realistic representation of how barrier disruption and repair evolve over time under pathological conditions.

Model performance depends on several technical parameters. The microfluidic perfusion rate and shear stress directly influence epithelial maturation, apical-basal polarity, brush border formation, and tight junction maintenance [149–151]. Chip architecture and membrane properties affect oxygen and nutrient exchange, cell adhesion, and interfacial signaling [150–152]. In addition, coculture with endothelial, stromal, or immune cells can improve the reconstruction of the intestinal barrier microenvironment. Functional evaluation should therefore not rely on morphology alone but should also include the transepithelial electrical resistance (TEER), paracellular tracer permeability, mucus-related readouts, and the expression and localization of tight junction proteins such as ZO-1, occludin, and claudins [153–154]. Compared with conventional tissue engineering approaches, organoid-on-a-chip systems offer greater physiological relevance, dynamic functional readouts, and better mechanistic resolution, which makes them particularly useful for studying complex barrier injury processes.

For example, Guo *et al.* established a SARS-CoV-2-induced intestinal response model using an intestinal organoid-on-a-chip system and reproduced viral infection-associated epithelial injury and inflammatory responses [155]. Morelli and colleagues induced barrier dysfunction by exposing the apical side of the intestinal epithelium to pro-inflammatory cytokines, including TNF-α, IL-1β, and IFN-γ, and further revealed the involvement of lipid signaling pathways in inflammation-driven barrier injury [156]. Trauma-related barrier injury has also begun to be studied in organoid-on-a-chip systems. Huang *et al.* developed a microfluidic intestinal organoid-on-a-chip model that reproduced the rapid oxygen dynamics of intestinal ischemia–reperfusion injury, a process closely related to post-traumatic and burn-associated intestinal barrier damage. This platform reproduced hypoxia–reoxygenation-induced metabolic disturbances, inflammatory responses, and epithelial injuries and enabled the identification of OLFM4 as a potential therapeutic target for intestinal ischemia–reperfusion injury [157]. Together, these studies show that organoid-on-a-chip systems not only reproduce disease phenotypes but also help define how inflammatory signaling, metabolic disturbance, trauma-related stress, and epithelial injury interact to drive barrier dysfunction.

### Drug screening and toxicological evaluation

Because orally administered drugs undergo intestinal absorption and often first-pass hepatic metabolism, gut-related organoid-on-a-chip platforms, particularly integrated gut–liver systems, are valuable for studying absorption, distribution, metabolism, excretion, and toxicity (ADMET) [69, 158–162]. In contrast to static single-organ models, organoid-on-a-chip technology allows dynamic exposure, fluid-mediated transport, and inter-organ coupling, which improves the prediction of pharmacokinetic and pharmacodynamic behaviors. This may be particularly relevant in severe trauma and burn settings, where intestinal barrier integrity and systemic drug handling are markedly altered [163–164]. Recent burn-related studies further support the translational value of advanced human-relevant platforms for therapeutic evaluation in this setting [164].

In studies of intestinal mucosal barrier dysfunction, organoid-on-a-chip platforms can be used not only to identify candidate compounds with anti-inflammatory, antioxidant, or barrier-restoring effects [165–167] but also to evaluate their mechanisms through functional barrier readouts. Compared with endpoint-based assays alone, simultaneous assessments of TEER, permeability changes, inflammatory mediator expressions, and tight junction protein expressions allow more comprehensive evaluations of therapeutic efficacy. These systems may therefore be particularly useful for preclinical drug screening and for detecting functional toxicities that are difficult to identify in conventional models.

Organoid-on-a-chip technology also provides a useful platform for studying intestinal nutrient absorption, transport, and metabolism [168–170]. By dynamically monitoring transepithelial nutrient flux together with barrier status, these systems may support the evaluation of nutrition-related interventions and the optimization of individualized nutritional strategies, particularly in disease states that are characterized by impaired barrier function [28, 169, 171].

### Regenerative medicine and precision medicine

Advances in organoid culture have also expanded the opportunities in regenerative medicine. Early regenerative approaches largely relied on directing embryonic or ASCs toward specific target cell types and tissues [172]. In contrast, organoid-based strategies offer a more tissue-relevant route for reconstruction and repair, and accumulating evidence supports the feasibility of organoid transplantation [48, 173]. For instance, Sugimoto and colleagues transplanted human ileal organoids into the mouse colon and observed the preservation of regional identity together with the formation of new villus-like structures [174]. Similar regenerative potential has been reported for organoids derived from the bile duct, retina, and heart [175–177]. In this context, organoid-on-a-chip technology is useful not only as a pre-transplantation culture platform but also as a controllable system for optimizing tissue maturation, barrier function, and adaptation to a host-like microenvironment. By modulating perfusion conditions, ECM support, and cellular composition, these platforms may improve the functional quality of graft-ready tissues and facilitate preclinical evaluation before transplantation.

The integration of patient-derived organoids, gene editing, and microfluidic engineering has also made organoid-on-a-chip technology highly relevant to precision medicine [141, 159, 178–179]. Patient-specific platforms can reproduce individual disease phenotypes and therapeutic responses *in vitro*, thereby supporting personalized drug testing and treatment selection.

Overall, organoid-on-a-chip technology has advanced the study of intestinal mucosal barrier dysfunction by linking functional reconstruction of the intestinal microenvironment with disease modeling, therapeutic evaluation, and translational investigation. The main advantages and limitations of these systems are summarized in Table 2. Broader adoption, however, remains limited by unresolved challenges in standardization, reproducibility, scalability, and translational readiness.

## Gut–organ–axis extensions

**Figure 4:**
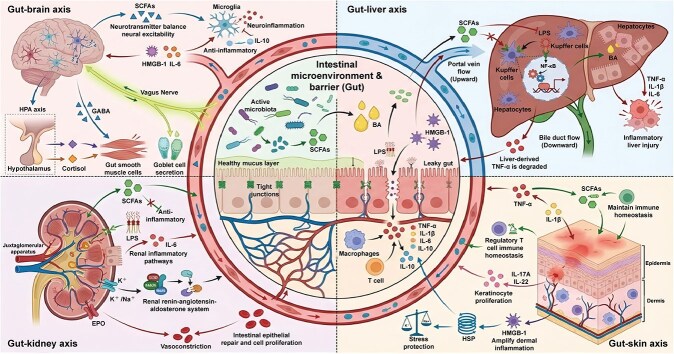
Gut-associated multi-organ crosstalk centered on the intestinal mucosal barrier. The intestinal mucosal barrier links microbiota-derived metabolites, inflammatory mediators, and bidirectional communication with distal organs. The figure summarizes the gut–liver, gut–brain, gut–skin, and gut–kidney axes, including protective effects of SCFAs, inflammatory signaling mediated by LPS and HMGB-1, and reciprocal feedback between the intestine and peripheral organs that influences barrier function, immune homeostasis, and systemic injury responses. *LPS* lipopolysaccharides, *HMGB-1* high mobility group box 1, *NF-κB* nuclear factor-kappa B, *TNF-α* tumor necrosis factor-alpha, *IL* interleukin, *BA* bile acids, *GABA* gamma-aminobutyric acid, *CA* catecholamines, *COR* cortisol, *HSP* heat shock protein, *RAAS* renin-angiotensin system, *EPO* erythropoietin

Building on their ability to reconstruct the intestinal barrier and model disease, intestinal organoid-on-a-chip systems have increasingly been extended to multi-organ platforms for studying distal physiological and pathological crosstalk. The gut is the largest immune organ and microbial ecosystem in the human body and communicates closely with other organs [180–186]. Because traditional cell-based and animal models have important limitations in modeling multi-organ interactions, intestinal organoid-on-a-chip systems provide a useful platform for studying gut-associated inter-organ crosstalk [21, 69]. Current multi-organ chip studies focus mainly on gut-associated axes such as the gut–liver, gut–brain, and gut–skin systems, along with other emerging gut-related platforms (Figure 4). Among them, the gut–liver axis is currently the most established, whereas the gut–brain and gut–skin axes place greater emphasis on distal signaling, inflammatory crosstalk, and systems-level integration.

### Gut–liver axis

Gut–liver–axis OoC systems that integrate intestinal and hepatic tissue modules and mainly model dynamic exchange and bidirectional crosstalk that are related to intestinal absorption, portal circulation, and hepatic metabolism are integrated [69, 158–161, 187–188]. Their applications include drug development and safety assessments [189], liver disease [190], gut–liver–axis disorders [142], and personalized medicine [159, 191–192]. These systems allow more precise evaluations of paracetamol hepatotoxicity and microbiota-mediated drug metabolism [161, 189] and support the investigation of gut–liver injury pathways that are induced by exogenous factors and disease states [109, 142, 190]. Among current gut–organ–axis models, the gut–liver axis is the most mature, supported by a relatively clear anatomical and physiological flow relationship and by its direct relevance to absorption, first-pass metabolism, and hepatointestinal toxicity. Its main strength lies in the functional coupling between intestinal exposure and the hepatic response. Its main limitation is the difficulty of reconciling tissue-specific medium requirements, multicellular complexity, and long-term stability.

### Gut–brain axis

A central focus of gut–brain–axis research is the altered permeability of the intestinal epithelial barrier and the blood–brain barrier (BBB) [185, 193]. OoC platforms have been used to establish gut–brain–axis models that reproduce physiological microenvironments, including gut–brain signaling and nutrient exchange [194]. These platforms have been used to study mechanisms relevant to gut–brain–axis disorders, such as IBS and neurodegenerative disease [194–198]. They also support investigations of gut–brain interaction mechanisms and drug toxicity screening, including that of antidepressants and gut microbiota modulators [188, 191, 197, 199–200]. This broad scope is valuable, but it also makes validation and causal interpretation more difficult. In many studies, the biological pathways linking intestinal events to neural outcomes remain only partly defined, and the relative contributions of immune, endocrine, metabolic, and microbial mediators are often difficult to separate. Gut–brain chips are therefore useful for hypothesis generation, but their translational interpretation still requires caution.

### Gut–skin axis

Evidence increasingly supports a physiological connection between the gut and the skin, and interest in the gut–skin axis has increased [182]. Lee *et al.* developed a modular intestine-skin dual-organ chip that allowed bidirectional signaling through continuous perfusion and provided a new platform for investigating gut-related factors in skin disease [201]. Their study revealed that impaired intestinal barrier function increased uptake of the free fatty acid palmitic acid (PA), thereby worsening its effects on skin cells. The model also showed increased levels of inflammatory factors and increased secretion of human β-defensin-2 (hBD-2), together with reduced skin cell viability [201]. Subsequent gut–microbiota–skin–axis chip studies have reproduced the inflammatory crosstalk between the gut and skin and helped clarify how the gut microbiota may influence skin health [202]. The gut–skin axis illustrates how intestinal dysfunction may affect peripheral epithelial tissues and distal inflammatory outcomes through circulating metabolites or inflammatory mediators. However, compared with gut–liver and gut–brain systems, this area is less mature, and many models remain proof-of-concept platforms rather than standardized disease models.

### Other gut–organ axis models

The gut interacts with many other organs, and related organoid-on-a-chip platforms continue to expand [69, 165, 203–205]. For example, acute lung injury and acute respiratory distress syndrome (ALI/ARDS) chip models have provided insight into gut-lung interactions [206–207]. An intestinal-kidney chip has been used to model bidirectional crosstalk in chronic kidney disease (CKD) [208]. Moreover, these systems highlight a common limitation: as the organ-level complexity increases, model controllability, medium compatibility, long-term stability, and mechanistic interpretability often become more difficult to maintain.

### Current bottlenecks in translation

Although organoid-based technologies are being increasingly recognized as promising alternatives to animal models, a lack of standardization and limited reproducibility remain major barriers to the broader use of organoid-on-a-chip systems [141, 209]. Key variables in organoid culture and chip operation, including the cellular microenvironment, matrix composition, differentiation state, biomechanical stimulation, chip architecture, and fabrication methods, are not yet standardized across laboratories, which leads to substantial variation in experimental results [210]. This problem is compounded by the biological heterogeneity in patient-derived organoids, including differences in tissue source, donor background, passage number, and maturation state, all of which can affect structural and functional readouts [210–211]. As a result, comparisons across studies remain difficult, and the confidence in mechanistic interpretations, drug-response evaluations, and translational assessments may be reduced [210–211]. Broader biomedical translation will therefore depend not only on expanding applications but also on improving reproducibility, engineering integration, and translational implementation at the platform level [141].

These issues are particularly important in studies of intestinal mucosal barrier dysfunction, where epithelial differentiation, inflammatory responsiveness, and tight junction integrity are highly sensitive to both biological and engineering conditions [35–36, 47, 143–146]. In trauma- and burn-related settings, patient heterogeneity and systemic inflammatory disturbance add further biological complexity, making it more difficult to distinguish disease-related phenotypes from model-derived variation and thereby weakening the robustness and translational value of experimental findings [35–36, 47, 143–146, 210–211].

A practical path toward standardization should include clearer criteria for organoid sourcing, expansion, and biobanking; chemically defined matrices and culture media to reduce batch-related variation; more consistent reporting of chip design, perfusion conditions, and biomechanical parameters; and consensus quality-control measures such as epithelial morphology, lineage marker expression, TEER, permeability, and tight junction-related readouts. Interlaboratory benchmarking studies and reporting guidelines will also be important for improving reproducibility and supporting regulatory acceptance [209–211].

Beyond standardization, broader application of organoid-on-a-chip technology remains limited by the complexity and manual nature of organoid culture and chip fabrication [212], which restrict scalability and make it difficult to meet the needs of high-throughput screening and industrial production. In addition, the lack of efficient, integrated, and reliable detection systems continues to hinder commercialization and broader translational use [213]. In response, next-generation intestinal organoid-on-a-chip platforms are being developed to improve robustness, automation, standardization, and clinical applicability.

### Next-generation intestinal organoid-on-a-chip

Next-generation intestinal organoid-on-a-chip platforms are being developed to address current limitations in standardization, scalability, and translational use through defined and animal-free culture systems, automated manufacturing, advanced fabrication methods, patient-derived resource platforms, and more complete reconstruction of biological complexity [72, 141, 214–215].

One major direction is the development of defined and animal-free culture systems. Conventional organoid culture still depends heavily on Matrigel and other poorly defined animal-derived matrices, which contribute to batch-to-batch variation and limit standardization [61]. Recent progress in fully synthetic and animal-free 3D culture substrates, with a growth efficiency comparable to that of Matrigel, represents an important step toward improving reproducibility, regulatory compatibility, and industrial scalability [61]. These chemically defined systems may also allow better control of matrix stiffness, biochemical signaling, and lineage-specific differentiation, thereby improving the robustness of intestinal organoid-on-a-chip platforms [61].

Another important direction is automated manufacturing. Organoid culture and chip fabrication remain labor-intensive and highly operator-dependent, which limits throughput and reproducibility [212]. Future production is likely to rely increasingly on robotic systems and programmable workflows that integrate chip design, assembly, dynamic culture, quality control, and data processing [216]. In this context, artificial intelligence (AI), particularly machine learning and deep learning, may help optimize protocols, support image-based monitoring, analyze multimodal datasets, and guide the control of culture conditions [213, 217–218]. AI-assisted approaches may help optimize cytokine supplementation schedules and fluid shear stress parameters, while real-time monitoring and rapid analytical feedback may further improve experimental consistency, reduce cost and labor, and support the development of high-throughput and patient-specific organoid-on-a-chip systems [219–220]. Broader implementation of AI in this field, however, will still depend on improved data standardization, shared databases, and interoperable analytical frameworks [213, 217–218].

The third major area is 3D bioprinting and advanced fabrication. Three-dimensional bioprinting offers a strategy for the rapid and customizable fabrication of microfluidic devices, engineered scaffolds, and spatially organized tissue constructs [221]. By allowing more precise control over the geometry, size, and cellular composition of organoid-associated structures, bioprinting may reduce batch-related variations and improve experimental reproducibility [214]. The integration of 3D bioprinting with hydrogel engineering has also created opportunities to construct vascularized intestinal organoid-on-a-chip patches for targeted tissue repair and transplantation [214]. These approaches may support not only more physiologically relevant *in vitro* models but also future regenerative applications that require scaffold-assisted implantation [214]. Current bioinks, however, still have important limitations in terms of biocompatibility, mechanical strength, and lineage-specific inductive capacity [222]. High-precision printing also often requires trade-offs between structural fidelity and cell viability, and differences in rheological properties and curing conditions across materials complicate process optimization [223–224].

Biobanks and patient-specific platforms are another important area. Large-scale organoid biobanks are becoming increasingly important for precision medicine, especially in oncology [225, 226–228]. In contrast, biobanks for non-neoplastic intestinal diseases remain limited [229–231]. Expanding disease-specific intestinal organoid biobanks could provide an important foundation for patient stratification, mechanistic studies, individualized drug screening, and rare disease research. For example, multi-omics analysis of Crohn’s disease organoid repositories has enabled the identification of clinically relevant disease subgroups and has reshaped classification frameworks for inflammatory bowel disease [231]. In the context of intestinal mucosal barrier dysfunction, such biobanks may serve not only as repositories of biological diversity but also as infrastructure for the standardization, benchmarking, and development of patient-specific organoid-on-a-chip platforms.

Future platforms will likely better integrate systemic immune responses, microbiota-associated signals, vascular interfaces, and multi-organ crosstalk, thereby more closely reproducing the pathophysiology of intestinal barrier dysfunction in complex disease states [72, 214–215]. This is particularly relevant in trauma and burn settings, where systemic inflammation, cytokine storm-like responses, and organ-organ interactions may strongly influence intestinal barrier injury and repair [35–36, 47, 143–146]. At present, however, incorporation of this degree of complexity into chip systems remains technically challenging, and the crosstalk across multi-organ platforms at different stages of disease progression still requires further study [72, 214–215]. In addition, for regenerative applications, the biosafety of residual undifferentiated cells in intestinal organoid transplantation remains an important concern that must be addressed before clinical use [10, 174–175, 232–233].

Taken together, these findings suggest that next-generation intestinal organoid-on-a-chip systems are evolving toward more defined, automated, and biologically integrated platforms. These advances may help address current limitations in standardization, scalability, and translational relevance while extending the use of organoid-on-a-chip technology in mechanistic research, drug development, regenerative medicine, and precision medicine. Technological progress alone, however, will not be sufficient for successful clinical translation, which will also require careful attention to ethical, regulatory, and societal considerations.

### Ethical and regulatory considerations

As organoid-on-a-chip technology moves closer to clinical use, ethical and regulatory issues require careful attention. Organoid research has advanced rapidly in recent years, but important ethical, legal, and societal questions have been raised [234–235]. One major issue is informed consent for donor-derived tissues, particularly because organoid systems may retain donor-specific biological features and may be expanded or reused in future studies [234, 236]. This concern is especially apparent in brain organoid research, where donors may be seen as having more lasting connection to their biological material [234]. Cross-species transplantation also raises questions about animal welfare and the boundary between human and non-human biological systems and therefore requires careful ethical review and evaluation of model suitability [237–239]. Another important concern is the premature clinical use of unproven organoid-based therapies, which could repeat problems seen previously with unregulated stem cell interventions [234, 240]. Responsible development of organoid-on-a-chip technology will therefore require strong ethical governance, appropriate regulatory oversight, and collaboration across multiple stakeholders to ensure scientifically rigorous, ethically responsible, and clinically credible translation [241].

## Conclusions

Intestinal organoid-on-a-chip technology has become a promising *in vitro* platform for intestinal research because it addresses a central challenge in the field: how to model the intestinal mucosal barrier as an integrated, dynamic, and clinically relevant system rather than as an isolated epithelial layer. By combining the tissue specificity of organoids with the controllability of microfluidic systems, these platforms allow more faithful reconstructions of barrier architectures, secretory functions, immune interactions, microbial crosstalk, and biomechanical regulation, and their applications now extend beyond barrier simulation to disease modeling, therapeutic evaluation, precision medicine, and studies of the gut–organ axis. Important challenges remain, including standardization, scalability, immune integration, and clinical translation, but continued progress across disciplines will likely support the development of more robust and clinically useful next-generation intestinal organoid-on-a-chip systems.

## Data Availability

The datasets used and analyzed during the current study are available from the corresponding author on reasonable request.

## References

[1] Sylvestre M, Di Carlo SE, Peduto L. Stromal regulation of the intestinal barrier. *Mucosal Immunol* 2023;16:221–31. 10.1016/j.mucimm.2023.01.006. 36708806 PMC10270662

[2] Untersmayr E, Brandt A, Koidl L, Bergheim I. The intestinal barrier dysfunction as driving factor of inflammaging. *Nutrients* 2022;14:949. 10.3390/nu14050949. 35267924 PMC8912763

[3] Mules TC, Inns S, Le Gros G. Helminths’ therapeutic potential to treat intestinal barrier dysfunction. *Allergy* 2023;78:2892–905. 10.1111/all.15812. 37449458

[4] Kartjito MS, Yosia M, Wasito E, Soloan G, Agussalim AF, Basrowi RW. Defining the relationship of gut microbiota, immunity, and cognition in early life-a narrative review. *Nutrients* 2023;15:2642. 10.3390/nu15122642. 37375546 PMC10304934

[5] Matar A, Damianos JA, Jencks KJ, Camilleri M. Intestinal barrier impairment, preservation, and repair: an update. *Nutrients* 2024;16:3494. 10.3390/nu16203494. 39458489 PMC11509958

[6] Macura B, Kiecka A, Szczepanik M. Intestinal permeability disturbances: causes, diseases and therapy. *Clin Exp Med* 2024;24:232. 10.1007/s10238-024-01496-9. 39340718 PMC11438725

[7] Damianos J, Abdelnaem N, Camilleri M. Gut goo: physiology, diet, and therapy of intestinal mucus and biofilms in gastrointestinal health and disease. *Clin Gastroenterol Hepatol Off Clin Pract J Am Gastroenterol Assoc* 2025;23:205–15. 10.1016/j.cgh.2024.09.007. PMC11761393 39426645

[8] Liu L, Yue Q, Chen J, Liu H, Zeng X. Intestinal injury signaling pathway in sepsis. *Front Immunol* 2025;16:1620965. 10.3389/fimmu.2025.1620965. 40655155 PMC12245687

[9] Ge P, Luo Y, Okoye CS, Chen H, Liu J, Zhang G, et al. Intestinal barrier damage, systemic inflammatory response syndrome, and acute lung injury: a troublesome trio for acute pancreatitis. *Biomed Pharmacother Biomedecine Pharmacother* 2020;132:110770. 10.1016/j.biopha.2020.110770. 33011613

[10] Wang M, Li R, Sheng S, Dong Z, Bai L, Wang X, et al. Combination therapy using intestinal organoids and their extracellular vesicles for inflammatory bowel disease complicated with osteoporosis. *J Orthop Translat* 2025;53:26–36. 10.1016/j.jot.2025.05.008. 40525095 PMC12167829

[11] Riedel S, Pheiffer C, Johnson R, Louw J, Muller CJF. Intestinal barrier function and immune homeostasis are missing links in obesity and type 2 diabetes development. *Front Endocrinol* 2021;12:833544. 10.3389/fendo.2021.833544. PMC8821109 35145486

[12] Kim S, Goel R, Kumar A, Qi Y, Lobaton G, Hosaka K, et al. Imbalance of gut microbiome and intestinal epithelial barrier dysfunction in patients with high blood pressure. *Clin Sci Lond Engl* 1979;2018:701–18. 10.1042/CS20180087. PMC5955695 29507058

[13] Abdullahi A, Amini-Nik S, Jeschke MG. Animal models in burn research. *Cell Mol LIFE Sci CMLS* 2014;71:3241–55. 10.1007/s00018-014-1612-5. 24714880 PMC4134422

[14] Kumari M, Alam K, Kaity S, Sah SK, Ravichandiran V, Roy S. Fabrication of multilayer heterogeneous cell assembly for pathophysiologically relevant 3Din-vitroIBD disease model for high throughput drug screening. *Biofabrication* 2025;17:2015. 10.1088/1758-5090/adc50e. 40132231

[15] Marín-Sáez J, Lopez-Ruiz R, Faria MA, Ferreira IMPLVO, Garrido FA. A comprehensive study on the digestion, absorption, and metabolization of tropane alkaloids in human cell models. *J Hazard Mater* 2024;480:136192. 10.1016/j.jhazmat.2024.136192. 39427354

[16] Hosic S, Lake W, Stas E, Koppes R, Breault DT, Murthy SK, et al. Cholinergic activation of primary human derived intestinal epithelium does not ameliorate TNF-α induced injury. *Cell Mol Bioeng* 2020;13:487–505. 10.1007/s12195-020-00633-0. 33184579 PMC7596162

[17] Rudolph SE, Longo BN, Tse MW, Houchin MR, Shokoufandeh MM, Chen Y, et al. Crypt-villus scaffold architecture for bioengineering functional human intestinal epithelium. *ACS Biomater Sci Eng* 2022;8:4942–55. 10.1021/acsbiomaterials.2c00851. 36191009 PMC10379436

[18] Mitrofanova O, Nikolaev M, Xu Q, Broguiere N, Cubela I, Camp JG, et al. Bioengineered human colon organoids with *in vivo*-like cellular complexity and function. *Cell Stem Cell* 2024;31:1175–1186.e7. 10.1016/j.stem.2024.05.007. 38876106

[19] Fey C, Truschel T, Nehlsen K, Damigos S, Horstmann J, Stradal T, et al. Enhancing pre-clinical research with simplified intestinal cell line models. *J Tissue Eng* 2024;15:20417314241228949. 10.1177/20417314241228949. 38449469 PMC10916479

[20] Li X-G, Chen M-X, Zhao S-Q, Wang X-Q. Intestinal models for personalized medicine: from conventional models to microfluidic primary intestine-on-a-chip. *Stem Cell Rev Rep* 2022;18:2137–51. 10.1007/s12015-021-10205-y. 34181185 PMC8237043

[21] Wu L, Ai Y, Xie R, Xiong J, Wang Y, Liang Q. Organoids/organs-on-a-chip: new frontiers of intestinal pathophysiological models. *Lab Chip* 2023;23:1192–212. 10.1039/d2lc00804a. 36644984

[22] Chelakkot C, Ghim J, Ryu SH. Mechanisms regulating intestinal barrier integrity and its pathological implications. *Exp Mol Med* 2018;50:1–9. 10.1038/s12276-018-0126-x. PMC6095905 30115904

[23] Stolfi C, Maresca C, Monteleone G, Laudisi F. Implication of intestinal barrier dysfunction in gut dysbiosis and diseases. *Biomedicines* 2022;10:289. 10.3390/biomedicines10020289. 35203499 PMC8869546

[24] Yuan S, Wang Q, Li J, Xue J-C, Li Y, Meng H, et al. Inflammatory bowel disease: an overview of chinese herbal medicine formula-based treatment. *Chin Med* 2022;17:74. 10.1186/s13020-022-00633-4. 35717380 PMC9206260

[25] Wang X, Ding C, Li H-B. The crosstalk between enteric nervous system and immune system in intestinal development, homeostasis and diseases. *Sci China Life Sci* 2024;67:41–50. 10.1007/s11427-023-2376-0. 37672184

[26] Li B, Yeganeh M, Lee D, Chusilp S, Balsamo F, Ganji N, et al. Exploring the complex pathophysiology of necrotizing enterocolitis in preterm neonates. *Annu Rev Pathol* 2025;21:37–58. 10.1146/annurev-pathmechdis-070224-014223. 40953322

[27] Chen S, Li T, Zhao P, Liang P, Huang X, Song L. et al. Stachyose alleviates high-fat diet-induced obesity via browning of white adipose tissue and modulation of gut microbiota. *Curr Res Food Sci* 2025;10:101081. 10.1016/j.crfs.2025.101081. 40485899 PMC12143653

[28] Chen X, Zhang P, Zhang Y, Fan S, Wei Y, Yang Z, et al. Potential effect of glutamine in the improvement of intestinal stem cell proliferation and the alleviation of burn-induced intestinal injury via activating YAP: a preliminary study. *Nutrients* 2023;15:1766. 10.3390/nu15071766. 37049605 PMC10097377

[29] He W, Wang Y. Intestinal barrier dysfunction in severe burn injury. *Burns Trauma* 2019;7:24. 10.1186/s41038-019-0162-3. 31372365 PMC6659221

[30] Breugelmans T, Oosterlinck B, Arras W, Ceuleers H, De Man J, Hold GL, et al. The role of mucins in gastrointestinal barrier function during health and disease. *Lancet Gastroenterol Hepatol* 2022;7:455–71. 10.1016/S2468-1253(21)00431-3. 35397245

[31] Okumura R, Takeda K. The role of the mucosal barrier system in maintaining gut symbiosis to prevent intestinal inflammation. *Semin Immunopathol* 2024;47:2. 10.1007/s00281-024-01026-5. 39589551 PMC11599372

[32] Kashihara H, Tanaka H, Kitamata M, Shiratsuchi G, Katsuno T, Tsukita K, et al. Functional landscape of mechanistic diversity in 27 claudin family members at tight junctions. *Sci Adv* 2025;11:eadx7431. 10.1126/sciadv.adx7431. 41171911 PMC12577684

[33] Naydenov NG, Hu G, Robak D, Zafar A, Makhmudova K, Lechuga S, et al. The septin cytoskeleton is a novel regulator of intestinal epithelial barrier integrity and mucosal inflammation. JCI. *Insight* 2025;10:e191538. 10.1172/jci.insight.191538. PMC12643519 41055961

[34] Martins-Gomes C, Nunes FM, Silva AM. Natural products as dietary agents for the prevention and mitigation of oxidative damage and inflammation in the intestinal barrier. *Antioxidants (Basel)* 2024;13:65. 10.3390/antiox13010065. 38247489 PMC10812469

[35] Zhang M, Liu Q, Meng H, Duan H, Liu X, Wu J, et al. Ischemia-reperfusion injury: molecular mechanisms and therapeutic targets. *Signal Transduct Target Ther* 2024;9:12. 10.1038/s41392-023-01688-x. 38185705 PMC10772178

[36] Chen Y, Han B, Guan X, Du G, Sheng B, Tang X, et al. Enteric fungi protect against intestinal ischemia-reperfusion injury via inhibiting the SAA1-GSDMD pathway. *J Adv Res* 2024;61:223–37. 10.1016/j.jare.2023.09.008. 37717911 PMC11258666

[37] Huang Y, Feng Y, Wang Y, Wang P, Wang F, Ren H. Severe burn-induced intestinal epithelial barrier dysfunction is associated with endoplasmic reticulum stress and autophagy in mice. *Front Physiol* 2018;9:441. 10.3389/fphys.2018.00441. 29740349 PMC5925571

[38] Palrasu M, Kakar K, Marudamuthu A, Hamida H, Thada S, Zhong Y, et al. AhR activation transcriptionally induces anti-microbial peptide alpha-defensin 1 leading to reversal of gut microbiota dysbiosis and colitis. *Gut Microbes* 2025;17:2460538. 10.1080/19490976.2025.2460538. 39894796 PMC11792800

[39] Zha X, Su S, Wu D, Zhang P, Wei Y, Fan S, et al. The impact of gut microbiota changes on the intestinal mucus barrier in burned mice: a study using 16S rRNA and metagenomic sequencing. Burns *Trauma* 2023;11:tkad056. 10.1093/burnst/tkad056. PMC10734567 38130728

[40] Peterson LW, Artis D. Intestinal epithelial cells: regulators of barrier function and immune homeostasis. *Nat Rev Immunol* 2014;14:141–53. 10.1038/nri3608. 24566914

[41] Shi N, Li N, Duan X, Niu H. Interaction between the gut microbiome and mucosal immune system. *Mil Med Res* 2017;4:14. 10.1186/s40779-017-0122-9. 28465831 PMC5408367

[42] Hays KE, Pfaffinger JM, Ryznar R. The interplay between gut microbiota, short-chain fatty acids, and implications for host health and disease. *Gut Microbes* 2024;16:2393270. 10.1080/19490976.2024.2393270. 39284033 PMC11407412

[43] Liu P, Wang Y, Yang G, Zhang Q, Meng L, Xin Y, et al. The role of short-chain fatty acids in intestinal barrier function, inflammation, oxidative stress, and colonic carcinogenesis. *Pharmacol Res* 2021;165:105420. 10.1016/j.phrs.2021.105420. 33434620

[44] Wu W-JH, Kim M, Chang L-C, Assie A, Saldana-Morales FB, Zegarra-Ruiz DF, et al. Interleukin-1β secretion induced by mucosa-associated gut commensal bacteria promotes intestinal barrier repair. *Gut Microbes* 2022;14:2014772. 10.1080/19490976.2021.2014772. 34989321 PMC8741296

[45] Bègue H, Ducreux A, Paradis T, Loiselet A, Mourer T, Lucchi G, et al. Candida albicans releases a peptide from the Rbt1 protein to promote its invasion into the gut epithelium. *Gut Microbes* 2025;17:2573038. 10.1080/19490976.2025.2573038. 41165448 PMC12578311

[46] Earley ZM, Akhtar S, Green SJ, Naqib A, Khan O, Cannon AR. et al. Burn injury alters the intestinal microbiome and increases gut permeability and bacterial translocation. *PLoS One* 2015;10:e0129996. 10.1371/journal.pone.0129996. 26154283 PMC4496078

[47] You X, Niu L, Fu J, Ge S, Shi J, Zhang Y, et al. Bidirectional regulation of the brain-gut-microbiota axis following traumatic brain injury. *Neural Regen Res* 2025;20:2153–68. 10.4103/NRR.NRR-D-24-00088. 39359076 PMC11759007

[48] Sato T, Vries RG, Snippert HJ, van de Wetering M, Barker N, Stange DE, et al. Single Lgr5 stem cells build crypt-villus structures *in vitro* without a mesenchymal niche. *Nature* 2009;459:262–5. 10.1038/nature07935. 19329995

[49] Lancaster MA, Renner M, Martin C-A, Wenzel D, Bicknell LS, Hurles ME, et al. Cerebral organoids model human brain development and microcephaly. *Nature* 2013;501:373–9. 10.1038/nature12517. 23995685 PMC3817409

[50] Verstegen MMA, Coppes RP, Beghin A, De Coppi P, Gerli MFM, de Graeff N, et al. Clinical applications of human organoids. *Nat Med* 2025;31:409–21. 10.1038/s41591-024-03489-3. 39901045

[51] Birtele M, Lancaster M, Quadrato G. Modelling human brain development and disease with organoids. *Nat Rev Mol Cell Biol* 2025;26:389–412. 10.1038/s41580-024-00804-1. 39668188

[52] Beumer J, Clevers H. Cell fate specification and differentiation in the adult mammalian intestine. *Nat Rev Mol Cell Biol* 2021;22:39–53. 10.1038/s41580-020-0278-0. 32958874

[53] Simpson HL, Smits E, Moerkens R, Wijmenga C, Mooiweer J, Jonkers IH, et al. Human organoids and organ-on-chips in coeliac disease research. *Trends Mol Med* 2025;31:117–37. 10.1016/j.molmed.2024.10.003. 39448329

[54] Kim S, Min S, Choi YS, Jo S-H, Jung JH, Han K, et al. Tissue extracellular matrix hydrogels as alternatives to matrigel for culturing gastrointestinal organoids. *Nat Commun* 2022;13:1692. 10.1038/s41467-022-29279-4. 35354790 PMC8967832

[55] Kaur S, Kaur I, Rawal P, Tripathi DM, Vasudevan A. Non-matrigel scaffolds for organoid cultures. *Cancer Lett* 2021;504:58–66. 10.1016/j.canlet.2021.01.025. 33582211

[56] Hughes CS, Postovit LM, Lajoie GA. Matrigel: a complex protein mixture required for optimal growth of cell culture. *Proteomics* 2010;10:1886–90. 10.1002/pmic.200900758. 20162561

[57] Gnecco JS, Brown A, Buttrey K, Ives C, Goods BA, Baugh L, et al. Organoid co-culture model of the human endometrium in a fully synthetic extracellular matrix enables the study of epithelial–stromal crosstalk. *Med N Y N* 2023;4:554–579.e9. 10.1016/j.medj.2023.07.004. PMC10878405 37572651

[58] Gjorevski N, Sachs N, Manfrin A, Giger S, Bragina ME, Ordóñez-Morán P, et al. Designer matrices for intestinal stem cell and organoid culture. *Nature* 2016;539:560–4. 10.1038/nature20168. 27851739

[59] Ye S, Boeter JWB, Mihajlovic M, van Steenbeek FG, van Wolferen ME, Oosterhoff LA, et al. A chemically defined hydrogel for human liver organoid culture. *Adv Funct Mater* 2020;30:2000893. 10.1002/adfm.202000893. 34658689 PMC7611838

[60] Jia Z, Wang Z. Photo-crosslinking hydrogel based on porcine small intestinal submucosa decellularized matrix/fish collagen/GelMA for culturing small intestinal organoids and repairing intestinal defects. *Int J Mol Sci* 2025;26:663. 10.3390/ijms26020663. 39859377 PMC11766382

[61] Wijnakker JJAPM, Lim S, Schreurs R, Faria JF, Korving J, Begthel H, et al. Invasin-functionalized PIC hydrogels enable long-term 3D culture of epithelial organoids. *Proc Natl Acad Sci U S A* 2025;122:e2507500122. 10.1073/pnas.2507500122. 41091766 PMC12557532

[62] Farin HF, Jordens I, Mosa MH, Basak O, Korving J, Tauriello DVF, et al. Visualization of a short-range wnt gradient in the intestinal stem-cell niche. *Nature* 2016;530:340–3. 10.1038/nature16937. 26863187

[63] Shi M, Fu P, Bonventre JV, McCracken KW. Directed differentiation of ureteric bud and collecting duct organoids from human pluripotent stem cells. *Nat Protoc* 2023;18:2485–508. 10.1038/s41596-023-00847-2. 37460630 PMC11154671

[64] Wang Y, Hou Q, Wu Y, Xu Y, Liu Y, Chen J, et al. Methionine deficiency and its hydroxy analogue influence chicken intestinal 3-dimensional organoid development. *Anim Nutr Zhongguo Xu Mu Shou Yi Xue Hui* 2022;8:38–51. 10.1016/j.aninu.2021.06.001. PMC8669257 34977374

[65] Hayashi R, Okubo T, Kudo Y, Ishikawa Y, Imaizumi T, Suzuki K, et al. Generation of 3D lacrimal gland organoids from human pluripotent stem cells. *Nature* 2022;605:126–31. 10.1038/s41586-022-04613-4. 35444274

[66] Xu H, Jiao D, Liu A, Wu K. Tumor organoids: applications in cancer modeling and potentials in precision medicine. *J Hematol OncolJ Hematol Oncol* 2022;15:58. 10.1186/s13045-022-01278-4. 35551634 PMC9103066

[67] Zhong G, Lin R, Jiang T, Huo H, Jiang J, Mu P, et al. Low dosage exposure of deoxynivalenol and copper synergistically enhanced the intestinal toxicity. *Environ Int* 2025;205:109891. 10.1016/j.envint.2025.109891. 41172574

[68] Rubert J, Schweiger PJ, Mattivi F, Tuohy K, Jensen KB, Lunardi A. Intestinal organoids: a tool for modelling diet-microbiome-host interactions. *Trends Endocrinol Metab TEM* 2020;31:848–58. 10.1016/j.tem.2020.02.004. 33086077

[69] Kim R, Sung JH. Recent advances in gut- and gut–organ-axis-on-a-chip models. *Adv Healthc Mater* 2024;13:e2302777. 10.1002/adhm.202302777. 38243887

[70] Zhao Y, Landau S, Okhovatian S, Liu C, Lu RXZ, Lai BFL, et al. Integrating organoids and organ-on-a-chip devices. *Nat Rev Bioeng* 2024;2:588–608. 10.1038/s44222-024-00207-z.

[71] Özkan A, LoGrande NT, Feitor JF, Goyal G, Ingber DE. Intestinal organ chips for disease modelling and personalized medicine. *Nat Rev Gastroenterol Hepatol* 2024;21:751–73. 10.1038/s41575-024-00968-3. 39192055

[72] Huang Y, Liu T, Huang Q, Wang Y. From organ-on-a-chip to human-on-a-chip: a review of research progress and latest applications. *ACS Sens* 2024;9:3466–88. 10.1021/acssensors.4c00004. 38991227

[73] Farhang Doost N, Srivastava SK. A comprehensive review of organ-on-a-chip technology and its applications. *Biosensors* 2024;14:225. 10.3390/bios14050225. 38785699 PMC11118005

[74] Thakar RG, Fenton KN. Bioethical implications of organ-on-a-chip on modernizing drug development. *Artif Organs* 2023;47:1553–8. 10.1111/aor.14620. 37578206 PMC10615722

[75] Frey N, Sönmez UM, Minden J, LeDuc P. Microfluidics for understanding model organisms. *Nat Commun* 2022;13:3195. 10.1038/s41467-022-30814-6. 35680898 PMC9184607

[76] Monteduro AG, Rizzato S, Caragnano G, Trapani A, Giannelli G, Maruccio G. Organs-on-chips technologies—a guide from disease models to opportunities for drug development. *Biosens Bioelectron* 2023;231:115271. 10.1016/j.bios.2023.115271. 37060819

[77] Orlova VV, Nahon DM, Cochrane A, Cao X, Freund C, van den Hil F, et al. Vascular defects associated with hereditary hemorrhagic telangiectasia revealed in patient-derived isogenic iPSCs in 3D vessels on chip. *Stem Cell Rep* 2022;17:1536–45. 10.1016/j.stemcr.2022.05.022. PMC9287680 35777360

[78] Wu J, Fang H, Zhang J, Yan S. Modular microfluidics for life sciences. *J Nanobiotechnology* 2023;21:85. 10.1186/s12951-023-01846-x. 36906553 PMC10008080

[79] Quintard C, Tubbs E, Jonsson G, Jiao J, Wang J, Werschler N, et al. A microfluidic platform integrating functional vascularized organoids-on-chip. *Nat Commun* 2024;15:1452. 10.1038/s41467-024-45710-4. 38365780 PMC10873332

[80] Chen H, Luo Z, Lin X, Zhu Y, Zhao Y. Sensors-integrated organ-on-a-chip for biomedical applications. *Nano Res* 2023;16:10072–10099. 10.1007/s12274-023-5651-9. PMC10130312 37359077

[81] Dabbagh Moghaddam F, Anvar A, Ilkhani E, Dadgar D, Rafiee M, Ranjbaran N, et al. Advances in engineering immune-tumor microenvironments on-a-chip: integrative microfluidic platforms for immunotherapy and drug discovery. *Mol Cancer* 2025;24:271. 10.1186/s12943-025-02479-4. 41152935 PMC12560574

[82] Zhu C, Wang K, Lv Y, Liu S, Shen Y, Zhang Y. Immobilization protocols of nanozyme with different morphologies in microfluidic chips for biosensing. *ACS Appl Mater Interfaces* 2025;17:57967–75. 10.1021/acsami.5c14204. 41060185

[83] Khetani S, Yong KW, Alhasadi MF, Hamedzadeh A, Shahsavari L, Rafiei A, et al. A programmable, 3D neuron-on-chip platform integrating near real-time biosensing and multiaxial loading for mechanobiological injury profiling. *Adv Sci Weinh Baden-Wurtt Ger* 2025;13:e10309. 10.1002/advs.202510309. PMC12786346 41058005

[84] Cao UMN, Zhang Y, Chen J, Sayson D, Pillai S, Tran SD. Microfluidic organ-on-a-chip: a guide to biomaterial choice and fabrication. *Int J Mol Sci* 2023;24:3232. 10.3390/ijms24043232. 36834645 PMC9966054

[85] Petta D, D’Amora U, D’Arrigo D, Tomasini M, Candrian C, Ambrosio L, et al. Musculoskeletal tissues-on-a-chip: role of natural polymers in reproducing tissue-specific microenvironments. *Biofabrication* 2022;14:2001. 10.1088/1758-5090/ac8767. 35931043

[86] Liu J, Du C, Chen J, Tang B, Liu S, Tan J, et al. Hydrogel microspheres empowering organ-on-a-chip systems: innovations and applications. *Small Weinh Bergstr Ger* 2025;21:e2504563. 10.1002/smll.202504563. 40685712

[87] Sharma A, Sances S, Workman MJ, Svendsen CN. Multi-lineage human iPSC-derived platforms for disease modeling and drug discovery. *Cell Stem Cell* 2020;26:309–29. 10.1016/j.stem.2020.02.011. 32142662 PMC7159985

[88] Lall D, Workman MJ, Sances S, Ondatje BN, Bell S, Lawless G, et al. An organ-chip model of sporadic ALS using iPSC-derived spinal cord motor neurons and an integrated blood–brain-like barrier. *Cell Stem Cell* 2025;32:1139–1153.e7. 10.1016/j.stem.2025.05.015. 40562033 PMC12233189

[89] Carter RD, Purnama U, Castro-Guarda M, Montes-Aparicio CN, Chandran A, Mbasu R, et al. Systems biology and functional assessments of human iPSC-cardiomyocyte models of insulin resistance capture key hallmarks of diabetic cardiomyopathy. *Diabetes* 2025;74:1929–45. 10.2337/db25-0204. 40956683 PMC12585165

[90] Mochida T, Miyoshi M, Kakinuma S, Shimizu T, Tsuchiya J, Watakabe K, et al. Crosstalk via ICAM-1 enhances supportive phenotype of stellate cells and drives hepatocyte proliferation in iPSC-derived hepatic organoids. *Stem Cell Rep* 2025;20:102642. 10.1016/j.stemcr.2025.102642. PMC12790731 40972589

[91] Kasendra M, Tovaglieri A, Sontheimer-Phelps A, Jalili-Firoozinezhad S, Bein A, Chalkiadaki A, et al. Development of a primary human small intestine-on-a-chip using biopsy-derived organoids. *Sci Rep* 2018;8:2871. 10.1038/s41598-018-21201-7. 29440725 PMC5811607

[92] Lindner M, Laporte A, Block S, Elomaa L, Weinhart M. Physiological shear stress enhances differentiation, mucus-formation and structural 3D organization of intestinal epithelial cells *in vitro*. *Cells* 2021;10:2062. 10.3390/cells10082062. 34440830 PMC8391940

[93] Lee KK, McCauley HA, Broda TR, Kofron MJ, Wells JM, Hong CI. Human stomach-on-a-chip with luminal flow and peristaltic-like motility. *Lab Chip* 2018;18:3079–85. 10.1039/c8lc00910d. 30238091 PMC6364752

[94] Lamers MM, van der Vaart J, Knoops K, Riesebosch S, Breugem TI, Mykytyn AZ. et al. An organoid-derived bronchioalveolar model for SARS-CoV-2 infection of human alveolar type II-like cells. *EMBO J* 2021;40:e105912. 10.15252/embj.2020105912. 33283287 PMC7883112

[95] Wang Z, Zhang Y, Li Z, Wang H, Li N, Deng Y. Microfluidic brain-on-a-chip: from key technology to system integration and application. *Small Weinh Bergstr Ger* 2023;19:e2304427. 10.1002/smll.202304427. 37653590

[96] Campisi M, Shin Y, Osaki T, Hajal C, Chiono V, Kamm RD. 3D self-organized microvascular model of the human blood–brain barrier with endothelial cells, pericytes and astrocytes. *Biomaterials* 2018;180:117–29. 10.1016/j.biomaterials.2018.07.014. 30032046 PMC6201194

[97] Zhi Y, Zhu Y, Wang J, Zhao J, Zhao Y. Cortical organoid-on-a-chip with physiological hypoxia for investigating tanshinone IIA-induced neural differentiation. *Res Wash DC* 2023;6:273. 10.34133/research.0273. PMC10907018 38434243

[98] Ma C, Banan Sadeghian R, Negoro R, Fujimoto K, Araoka T, Ishiguro N, et al. Protocol to develop a proximal tubule-on-chip model based on hiPSC-derived kidney organoids for functional analysis of renal transporters. *STAR Protoc* 2025;6:103777. 10.1016/j.xpro.2025.103777. 40266846 PMC12225029

[99] Brandenberg N, Hoehnel S, Kuttler F, Homicsko K, Ceroni C, Ringel T, et al. High-throughput automated organoid culture via stem-cell aggregation in microcavity arrays. *Nat Biomed Eng* 2020;4:863–74. 10.1038/s41551-020-0565-2. 32514094

[100] Shrestha S, Lekkala VKR, Acharya P, Kang S-Y, Vanga MG, Lee M-Y. Reproducible generation of human liver organoids (HLOs) on a pillar plate platform via microarray 3D bioprinting. *Lab Chip* 2024;24:2747–61. 10.1039/d4lc00149d. 38660778 PMC11605706

[101] Fumadó Navarro J, Crilly S, Chan WK, Browne S, Mason JO, Vallejo-Giraldo C, et al. Cerebral organoids with integrated endothelial networks emulate the neurovascular unit and mitigate core necrosis. *Adv Sci Weinh Baden-Wurtt Ger* 2025;12:e07256. 10.1002/advs.202507256. PMC12631921 40884248

[102] Yu T, Yang Q, Peng B, Gu Z, Zhu D. Vascularized organoid-on-a-chip: design, imaging, and analysis. *Angiogenesis* 2024;27:147–72. 10.1007/s10456-024-09905-z. 38409567

[103] Li B, Tang Y, Huang Z, Ma L, Song J, Xue L. Synergistic innovation in organ-on-a-chip and organoid technologies: reshaping the future of disease modeling, drug development and precision medicine. Protein. *Cell* 2025;:1-18. 10.1093/procel/pwaf058. PMC13429468 40653861

[104] Ramadan Q, Jing L. Characterization of tight junction disruption and immune response modulation in a miniaturized caco-2/U937 coculture-based *in vitro* model of the human intestinal barrier. *Biomed Microdevices* 2016;18:11. 10.1007/s10544-016-0035-5. 26809386

[105] Sibilio S, Mennella R, Gregorio VD, Rocca AL, Urciuolo F, Imparato G, et al. A novel membrane-on-chip guides morphogenesis for the reconstruction of the intestinal crypt-villus axis. *Biofabrication* 2024;16:5019. 10.1088/1758-5090/ad6599. 39029501

[106] Jang Y, Kim H, Oh J. An array of carbon nanofiber Bundle_Based 3D *in vitro* intestinal microvilli for mimicking functional and physical activities of the small intestine. *Small Weinh Bergstr Ger* 2024;20:e2404842. 10.1002/smll.202404842. 39212639

[107] Floor E, Su J, Chatterjee M, Kuipers ES, IJssennagger N, Heidari F, et al. Development of a caco-2-based intestinal mucosal model to study intestinal barrier properties and bacteria-mucus interactions. *Gut Microbes* 2025;17:2434685. 10.1080/19490976.2024.2434685. 39714032 PMC11702969

[108] Sontheimer-Phelps A, Chou DB, Tovaglieri A, Ferrante TC, Duckworth T, Fadel C, et al. Human colon-on-a-chip enables continuous *in vitro* analysis of colon mucus layer accumulation and physiology. *Cell Mol Gastroenterol Hepatol* 2020;9:507–26. 10.1016/j.jcmgh.2019.11.008. 31778828 PMC7036549

[109] Almalla A, Alzain N, Elomaa L, Richter F, Scholz J, Lindner M, et al. Hydrogel-integrated millifluidic systems: advancing the fabrication of mucus-producing human intestinal models. *Cells* 2024;13:1080. 10.3390/cells13131080. 38994934 PMC11240340

[110] Kharaghani D, DeLoid GM, He P, Swenor B, Bui TH, Zuverza-Mena N, et al. Toxicity and absorption of polystyrene micro-nanoplastics in healthy and crohn’s disease human duodenum-chip models. *J Hazard Mater* 2025;490:137714. 10.1016/j.jhazmat.2025.137714. 40022921 PMC12051489

[111] Feile A, Wegner VD, Raasch M, Mosig AS. Immunocompetent intestine-on-chip model for analyzing gut mucosal immune responses. *J Vis Exp JoVE* 2024;207:e66603. 10.3791/66603. 38856194

[112] Gijzen L, Marescotti D, Raineri E, Nicolas A, Lanz HL, Guerrera D, et al. An intestine-on-a-chip model of plug-and-play modularity to study inflammatory processes. *SLAS Technol* 2020;25:585–97. 10.1177/2472630320924999. 32576063 PMC7684793

[113] Min S, Than N, Shin YC, Hu G, Shin W, Ambrosini YM, et al. Live probiotic bacteria administered in a pathomimetic leaky gut chip ameliorate impaired epithelial barrier and mucosal inflammation. *Sci Rep* 2022;12:22641. 10.1038/s41598-022-27300-w. 36587177 PMC9805460

[114] Penarete-Acosta D, Mittal M, Chakraborty S, Han A, Jayaraman A. Interplay between dietary fiber, macrophages and colonocytes in a microfluidic model of host-microbiota interactions in colorectal cancer. *Lab Chip* 2025;25:5482–96. 10.1039/d5lc00052a. 40994427

[115] Grassart A, Malardé V, Gobaa S, Sartori-Rupp A, Kerns J, Karalis K, et al. Bioengineered human organ-on-chip reveals intestinal microenvironment and mechanical forces impacting shigella infection. *Cell Host Microbe* 2019;26:435–444.e4. 10.1016/j.chom.2019.08.007. 31492657

[116] Trbojević-Stanković JB, Milićević NM, Milosević DP, Despotović N, Davidović M, Erceg P, et al. Morphometric study of healthy jejunal and ileal mucosa in adult and aged subjects. *Histol Histopathol* 2010;25:153–8. 10.14670/HH-25.153. 20017102

[117] Yi B, Shim KY, Ha SK, Han J, Hoang H-H, Choi I, et al. Three-dimensional *in vitro* gut model on a villi-shaped collagen scaffold. *BioChip J* 2017;11:219–31. 10.1007/s13206-017-1307-8.

[118] Costello CM, Hongpeng J, Shaffiey S, Yu J, Jain NK, Hackam D, et al. Synthetic small intestinal scaffolds for improved studies of intestinal differentiation. *Biotechnol Bioeng* 2014;111:1222–32. 10.1002/bit.25180. 24390638 PMC4233677

[119] Wang Y, Gunasekara DB, Reed MI, DiSalvo M, Bultman SJ, Sims CE, et al. A microengineered collagen scaffold for generating a polarized crypt-villus architecture of human small intestinal epithelium. *Biomaterials* 2017;128:44–55. 10.1016/j.biomaterials.2017.03.005. 28288348 PMC5392043

[120] Altay G, Tosi S, García-Díaz M, Martínez E. Imaging the cell morphological response to 3D topography and curvature in engineered intestinal tissues. *Front Bioeng Biotechnol* 2020;8:294. 10.3389/fbioe.2020.00294. 32318564 PMC7154059

[121] Gustafsson JK, Johansson MEV. The role of goblet cells and mucus in intestinal homeostasis. *Nat Rev Gastroenterol Hepatol* 2022;19:785–803. 10.1038/s41575-022-00675-x. 36097076

[122] Ojo BA, VanDussen KL, Rosen MJ. The promise of patient-derived colon organoids to model ulcerative colitis. *Inflamm Bowel Dis* 2022;28:299–308. 10.1093/ibd/izab161. 34251431 PMC8804507

[123] Ren J, Huang S. Intestinal organoids in inflammatory bowel disease: advances, applications, and future directions. *Front Cell Dev Biol* 2025;13:1517121. 10.3389/fcell.2025.1517121. 40421006 PMC12104276

[124] Hahn S, Han IW, Shin SH, Kim G, Kim JH. Modeling diabetic intestinal organoids: aspects of rapid gut barrier disruption. *Biochem Biophys Res Commun* 2025;760:151730. 10.1016/j.bbrc.2025.151730. 40168710

[125] Huang S, Zhang S, Chen L, Pan X, Wen Z, Chen Y, et al. Lipopolysaccharide induced intestinal epithelial injury: a novel organoids-based model for sepsis *in vitro*. *Chin Med J* 2022;135:2232–9. 10.1097/CM9.0000000000002348. 36355867 PMC9771316

[126] Zhang M, Huang X, Zhang Y, Yu M, Yuan X, Xu Y, et al. Gut microbial metabolite butyrate suppresses hepatocellular carcinoma growth via CXCL11-dependent enhancement of natural killer cell infiltration. *Gut Microbes* 2025;17:2519706. 10.1080/19490976.2025.2519706. 40576244 PMC12218501

[127] Nearing JT, Douglas GM, Hayes MG, MacDonald J, Desai DK, Allward N, et al. Microbiome differential abundance methods produce different results across 38 datasets. *Nat Commun* 2022;13:342. 10.1038/s41467-022-28034-z. 35039521 PMC8763921

[128] Wang H, Li X, Shi P, You X, Zhao G. Establishment and evaluation of on-chip intestinal barrier biosystems based on microfluidic techniques. *Mater Today Bio* 2024;26:101079. 10.1016/j.mtbio.2024.101079. PMC11107260 38774450

[129] Kollmann C, Buerkert H, Meir M, Richter K, Kretzschmar K, Flemming S, et al. Human organoids are superior to cell culture models for intestinal barrier research. *Front Cell Dev Biol* 2023;11:1223032. 10.3389/fcell.2023.1223032. 37849736 PMC10577213

[130] Palamà MEF, Aiello M, Borka G, Furci J, Parodi I, Firpo G, et al. A dynamic double-flow gut-on-chip model for predictive absorption studies *in vitro*. *Adv Mater Technol* 2025;10:2401661. 10.1002/admt.202401661.

[131] Li Y, Tang W, Guo M. The cell as matter: connecting molecular biology to cellular functions. *Matter* 2021;4:1863–91. 10.1016/j.matt.2021.03.013. 35495565 PMC9053450

[132] Tang W, Huang J, Pegoraro AF, Zhang JH, Tang Y, Kotton DN, et al. Topology and nuclear size determine cell packing on growing lung spheroids. *Phys Rev X* 2025;15:11067. 10.1103/physrevx.15.011067. PMC12122012 40444063

[133] Virumbrales-Muñoz M, Ayuso JM, Gong MM, Humayun M, Livingston MK, Lugo-Cintrón KM. et al. Microfluidic lumen-based systems for advancing tubular organ modeling. *Chem Soc Rev* 2020;49:6402–42. 10.1039/d0cs00705f. 32760967 PMC7521761

[134] Li Z, Hui J, Yang P, Mao H. Microfluidic organ-on-a-chip system for disease modeling and drug development. *Biosensors* 2022;12:370. 10.3390/bios12060370. 35735518 PMC9220862

[135] Wang X, Stefanello ST, Shahin V, Qian Y. From mechanoelectric conversion to tissue regeneration: translational progress in piezoelectric materials. *Adv Mater Deerfield Beach Fla* 2025;37:2417564. 10.1002/adma.202417564. PMC12369703 40434211

[136] Pérez-González C, Ceada G, Greco F, Matejčić M, Gómez-González M, Castro N, et al. Mechanical compartmentalization of the intestinal organoid enables crypt folding and collective cell migration. *Nat Cell Biol* 2021;23:745–57. 10.1038/s41556-021-00699-6. 34155382 PMC7611697

[137] Xue S-L, Yang Q, Liberali P, Hannezo E. Mechanochemical bistability of intestinal organoids enables robust morphogenesis. *Nat Phys* 2025;21:608–17. 10.1038/s41567-025-02792-1. 40248571 PMC11999871

[138] Baghdadi MB, Houtekamer RM, Perrin L, Rao-Bhatia A, Whelen M, Decker L, et al. PIEZO-dependent mechanosensing is essential for intestinal stem cell fate decision and maintenance. *Science* 2024;386:eadj7615. 10.1126/science.adj7615. 39607940

[139] Bao L, Cui X, Wang X, Wu J, Guo M, Yan N. et al. Carbon nanotubes promote the development of intestinal organoids through regulating extracellular matrix viscoelasticity and intracellular energy metabolism. *ACS Nano* 2021;15:15858–73. 10.1021/acsnano.1c03707. 34622660

[140] Zhou Y, Wang F, Zhao R, Huang T, Su W, Wen Y, et al. Advanced strategies in organoid/organ-on-a-chip for food safety and nutrition. *Food Res Int* 2025;219:117011. 10.1016/j.foodres.2025.117011. 40922158

[141] Wang H, Ning X, Zhao F, Zhao H, Li D. Human organoids-on-chips for biomedical research and applications. *Theranostics* 2024;14:788–818. 10.7150/thno.90492. 38169573 PMC10758054

[142] Yilmaz EG, Hacıosmanoğlu N, Jordi SBU, Yilmaz B, Inci F. Revolutionizing IBD research with on-chip models of disease modeling and drug screening. *Trends Biotechnol* 2025;43:1062–78. 10.1016/j.tibtech.2024.10.002. 39523166

[143] Villablanca EJ, Selin K, Hedin CRH. Mechanisms of mucosal healing: treating inflammatory bowel disease without immunosuppression? *Nat Rev Gastroenterol Hepatol* 2022;19:493–507. 10.1038/s41575-022-00604-y. 35440774

[144] Lin J, Wei Y, Gu X, Liu M, Wang M, Zhou R, et al. Nanotherapeutics-mediated restoration of pancreatic homeostasis and intestinal barrier for the treatment of severe acute pancreatitis. *J Control Release Off J Control Release Soc* 2025;377:93–105. 10.1016/j.jconrel.2024.11.022. 39542256

[145] Huang Y, Zhou J, Wang S, Xiong J, Chen Y, Liu Y, et al. Indoxyl sulfate induces intestinal barrier injury through IRF1-DRP1 axis-mediated mitophagy impairment. *Theranostics* 2020;10:7384–400. 10.7150/thno.45455. 32641998 PMC7330852

[146] Zhao L, Huang Y, Ye Z, Chen W, Zhang N, Wen Z, et al. Short-chain fatty acids attenuate sepsis-induced gut dysbiosis and hippocampal neuroinflammation via NLRP6 inflammasome activation in mice. *Int J Surg Lond Engl* 2025;112:570–582. 10.1097/JS9.0000000000003554. PMC12825554 41159411

[147] Lechuga S, Braga-Neto MB, Naydenov NG, Rieder F, Ivanov AI. Understanding disruption of the gut barrier during inflammation: should we abandon traditional epithelial cell lines and switch to intestinal organoids? *Front Immunol* 2023;14:1108289. 10.3389/fimmu.2023.1108289. 36875103 PMC9983034

[148] Günther C, Winner B, Neurath MF, Stappenbeck TS. Organoids in gastrointestinal diseases: from experimental models to clinical translation. *Gut* 2022;71:1892–908. 10.1136/gutjnl-2021-326560. 35636923 PMC9380493

[149] Mennella R, Sibilio S, Urciuolo F, Imparato G, Netti PA. Flow-induced mechano-modulation of intestinal permeability on chip. *Mater Today Bio* 2025;33:101951. 10.1016/j.mtbio.2025.101951. PMC12180969 40547488

[150] Wang X, Zhu Y, Cheng Z, Zhang C, Liao Y, Liu B, et al. Emerging microfluidic gut-on-a-chip systems for drug development. *Acta Biomater* 2024;188:48–64. 10.1016/j.actbio.2024.09.012. 39299625

[151] Keuper-Navis M, Eslami Amirabadi H, Donkers J, Walles M, Poller B, Heming B, et al. Intestinal cells-on-chip for permeability studies. *Micromachines* 2024;15:1464. 10.3390/mi15121464. 39770217 PMC11679574

[152] Papamichail L, Koch LS, Veerman D, Broersen K, van der Meer AD. Organoids-on-a-chip: microfluidic technology enables culture of organoids with enhanced tissue function and potential for disease modeling. *Front Bioeng Biotechnol* 2025;13:1515340. 10.3389/fbioe.2025.1515340. PMC11933005 40134772

[153] Malik M, Steele SA, Mitra D, Long CJ, Hickman JJ. Trans-epithelial/endothelial electrical resistance (TEER): current state of integrated TEER measurements in organ-on-a-chip devices. *Curr Opin Biomed Eng* 2025;34:100588. 10.1016/j.cobme.2025.100588. 40276329 PMC12017418

[154] Holzreuter MA, Segerink LI. Innovative electrode and chip designs for transendothelial electrical resistance measurements in organs-on-chips. *Lab Chip* 2024;24:1121–34. 10.1039/d3lc00901g. 38165817 PMC10898416

[155] Guo Y, Luo R, Wang Y, Deng P, Song T, Zhang M, et al. SARS-CoV-2 induced intestinal responses with a biomimetic human gut-on-chip. *Sci Bull* 2021;66:783–93. 10.1016/j.scib.2020.11.015. PMC7704334 33282445

[156] Morelli M, Savova MV, Queiroz K, Harms AC, Hankemeier T. Cytokine-induced barrier dysfunction and lipid signaling in a gut-on-chip model. *FASEB J Off Publ Fed Am Soc Exp Biol* 2025;39:e71059. 10.1096/fj.202501685R. PMC12510296 41065754

[157] Huang J, Xu Z, Jiao J, Li Z, Li S, Liu Y, et al. Microfluidic intestinal organoid-on-a-chip uncovers therapeutic targets by recapitulating oxygen dynamics of intestinal ischemia/reperfusion injury. *Bioact Mater* 2023;30:1–14. 10.1016/j.bioactmat.2023.07.001. 37534235 PMC10391666

[158] Hu W, Wang Y, Han J, Zhang W, Chen J, Li X, et al. Microfluidic organ-on-a-chip models for the gut–liver axis: from structural mimicry to functional insights. *Biomater Sci* 2025;13:1624–56. 10.1039/d4bm01273a. 40019226

[159] Gough A, Soto-Gutierrez A, Vernetti L, Ebrahimkhani MR, Stern AM, Taylor DL. Human biomimetic liver microphysiology systems in drug development and precision medicine. *Nat Rev Gastroenterol Hepatol* 2021;18:252–68. 10.1038/s41575-020-00386-1. 33335282 PMC9106093

[160] Mehta V, Karnam G, Madgula V. Liver-on-chips for drug discovery and development. *Mater Today Bio* 2024;27:101143. 10.1016/j.mtbio.2024.101143. PMC11279310 39070097

[161] Lucchetti M, Aina KO, Grandmougin L, Jäger C, Pérez Escriva P, Letellier E, et al. An organ-on-chip platform for simulating drug metabolism along the gut–liver axis. *Adv Healthc Mater* 2024;13:e2303943. 10.1002/adhm.202303943. 38452399

[162] Alqahtani S, Bukhari I, Albassam A, Alenazi M. An update on the potential role of intestinal first-pass metabolism for the prediction of drug-drug interactions: the role of PBPK modeling. *Expert Opin Drug Metab Toxicol* 2018;14:625–34. 10.1080/17425255.2018.1482277. 29806951

[163] Guo Y, Xie Y, Qin J. A generic pump-free organ-on-a-chip platform for assessment of intestinal drug absorption. *Biotechnol J* 2024;19:e2300390. 10.1002/biot.202300390. 38375564

[164] Song Y, Li Y, Hu W, Li F, Sheng H, Huang C, et al. Luminol-conjugated cyclodextrin biological nanoparticles for the treatment of severe burn-induced intestinal barrier disruption. *Burns Trauma* 2024;12:tkad054. 10.1093/burnst/tkad054. 38444636 PMC10910847

[165] Ding X, Xu N, Zhang W, Wang P. Integrated microfluidic three-organ chip for real-time toxicity analysis of fluorotelomer alcohols in the gut-vascular-nerve axis. *Lab Chip* 2025;25:6170–6. 10.1039/d5lc00631g. 41082182

[166] Azar J, Bahmad HF, Daher D, Moubarak MM, Hadadeh O, Monzer A, et al. The use of stem cell-derived organoids in disease modeling: an update. *Int J Mol Sci* 2021;22:7667. 10.3390/ijms22147667. 34299287 PMC8303386

[167] Pat Y, Yazici D, Zeyneloglu C, Babayev H, Ardicli S, Garci-Sanchez A, et al. Cellular stress, inflammation and barrier damage in gut epithelial cells caused by aspartame. *Allergy* 2025;81:884–901. 10.1111/all.70095. PMC12954558 41137210

[168] Rodrigues DB, Failla ML. Intestinal cell models for investigating the uptake, metabolism and absorption of dietary nutrients and bioactive compounds. *Curr Opin Food Sci* 2021;41:169–79. 10.1016/j.cofs.2021.04.002.

[169] Wang X, Xia Y, Wang J, Zhang Y, Lv P, Wang Y. Intestinal organoids as advanced modeling platform for food research: a review. *Crit Rev Food Sci Nutr* 2025;66:1463–1481. 10.1080/10408398.2025.2546510. 40838702

[170] Bein A, Fadel CW, Swenor B, Cao W, Powers RK, Camacho DM, et al. Nutritional deficiency in an intestine-on-a-chip recapitulates injury hallmarks associated with environmental enteric dysfunction. *Nat Biomed Eng* 2022;6:1236–47. 10.1038/s41551-022-00899-x. 35739419 PMC9652151

[171] Nie H-Y, Ge J, Huang G-X, Liu K-G, Yue Y, Li H, et al. New insights into the intestinal barrier through “gut–organ” axes and a glimpse of the microgravity’s effects on intestinal barrier. *Front Physiol* 2024;15:1465649. 10.3389/fphys.2024.1465649. 39450142 PMC11499591

[172] Wu X, Wang S, Li M, Li J, Shen J, Zhao Y, et al. Conditional reprogramming: next generation cell culture. *Acta Pharm Sin B* 2020;10:1360–81. 10.1016/j.apsb.2020.01.011. 32963937 PMC7488362

[173] Tang X-Y, Wu S, Wang D, Chu C, Hong Y, Tao M, et al. Human organoids in basic research and clinical applications. *Signal Transduct Target Ther* 2022;7:168. 10.1038/s41392-022-01024-9. 35610212 PMC9127490

[174] Sugimoto S, Kobayashi E, Fujii M, Ohta Y, Arai K, Matano M, et al. An organoid-based organ-repurposing approach to treat short bowel syndrome. *Nature* 2021;592:99–104. 10.1038/s41586-021-03247-2. 33627870

[175] Sampaziotis F, Muraro D, Tysoe OC, Sawiak S, Beach TE, Godfrey EM, et al. Cholangiocyte organoids can repair bile ducts after transplantation in the human liver. *Science* 2021;371:839–46. 10.1126/science.aaz6964. 33602855 PMC7610478

[176] Ozaki A, Sakai D, Mandai M. hPSC-based treatment of retinal diseases—current progress and challenges. *Adv Drug Deliv Rev* 2025;221:115587. 10.1016/j.addr.2025.115587. 40228605

[177] Hou Y, Cao C, Guo J, Du P, Li D, Yuan P, et al. From the laboratory to the clinic: differentiation, disease modeling and transplantation of cardiac organoids. *Adv Healthc Mater* 2025;15:e02698. 10.1002/adhm.202502698. 41147803

[178] Zhou C, Li Z, Lu K, Liu Y, Xuan L, Mao H, et al. Advances in human organs-on-chips and applications for drug screening and personalized medicine. *Fundam Res* 2025;5:1258–72. 10.1016/j.fmre.2023.12.019. 40528968 PMC12167889

[179] Ingber DE . Human organs-on-chips for disease modelling, drug development and personalized medicine. *Nat Rev Genet* 2022;23:467–91. 10.1038/s41576-022-00466-9. 35338360 PMC8951665

[180] Tilg H, Adolph TE, Trauner M. Gut–liver axis: pathophysiological concepts and clinical implications. *Cell Metab* 2022;34:1700–18. 10.1016/j.cmet.2022.09.017. 36208625

[181] Meerschaert KA, Chiu IM. The gut–brain axis and pain signalling mechanisms in the gastrointestinal tract. *Nat Rev Gastroenterol Hepatol* 2025;22:206–21. 10.1038/s41575-024-01017-9. 39578592 PMC13737996

[182] Zhao Y, Yu C, Zhang J, Yao Q, Zhu X, Zhou X. The gut–skin axis: emerging insights in understanding and treating skin diseases through gut microbiome modulation (review). *Int J Mol Med* 2025;56:210. 10.3892/ijmm.2025.5651. 41041846 PMC12494302

[183] Yang T, Richards EM, Pepine CJ, Raizada MK. The gut microbiota and the brain–gut–kidney axis in hypertension and chronic kidney disease. *Nat Rev Nephrol* 2018;14:442–56. 10.1038/s41581-018-0018-2. 29760448 PMC6385605

[184] Liu Q, Ruan K, An Z, Li L, Ding C, Xu D, et al. Updated review of research on the role of the gut microbiota and microbiota-derived metabolites in acute pancreatitis progression and inflammation-targeted therapy. *Int J Biol Sci* 2025;21:1242–58. 10.7150/ijbs.108858. 39897025 PMC11781165

[185] Ohara TE, Hsiao EY. Microbiota-neuroepithelial signalling across the gut–brain axis. *Nat Rev Microbiol* 2025;23:371–84. 10.1038/s41579-024-01136-9. 39743581

[186] Ramasamy B, Sharma DK, Callary SA, Ramadass B, Solomon LB, Atkins GJ. Role of gut microbiota disruption in prosthetic joint infection: a scoping review. *Lancet Microbe* 2025;6:101193. 10.1016/j.lanmic.2025.101193. 40934939

[187] Zhang Y, Liu Y, Liang X, Wen Y, Zhao J, He Y, et al. Intestinal barrier in chronic gut and liver diseases: pathogenesis and therapeutic targets. *Acta Pharm Sin B* 2025;15:5515–36. 10.1016/j.apsb.2025.08.028. 41311385 PMC12648041

[188] Kang SG, Choi YY, Mo SJ, Kim TH, Ha JH, Hong DK, et al. Effect of gut microbiome-derived metabolites and extracellular vesicles on hepatocyte functions in a gut–liver. *Nano Converg* 2023;10:5. 10.1186/s40580-022-00350-6. 36645561 PMC9842828

[189] Marin TM, de Carvalho IN, Rocco SA, Basei FL, de Carvalho M, de Almeida GK, et al. Acetaminophen absorption and metabolism in an intestine/liver microphysiological system. *Chem Biol Interact* 2019;299:59–76. 10.1016/j.cbi.2018.11.010. 30496738

[190] Liu H, Yin G, Kohlhepp MS, Schumacher F, Hundertmark J, Hassan MIA, et al. Dissecting acute drug-induced hepatotoxicity and therapeutic responses of steatotic liver disease using primary mouse liver and blood cells in a liver-on-a-chip model. *Adv Sci Weinh Baden-Wurtt Ger* 2024;11:e2403516. 10.1002/advs.202403516. PMC11321671 38868948

[191] Kim M-H, van Noort D, Sung JH, Park S. Organ-on-a-chip for studying gut–brain interaction mediated by extracellular vesicles in the gut microenvironment. *Int J Mol Sci* 2021;22:13513. 10.3390/ijms222413513. 34948310 PMC8707342

[192] Góralska J, Raźny U, Polus A, Dziewońska A, Gruca A, Zdzienicka A, et al. Enhanced GIP secretion in obesity is associated with biochemical alteration and miRNA contribution to the development of liver steatosis. *Nutrients* 2020;12:476. 10.3390/nu12020476. 32069846 PMC7071278

[193] Mulay AR, Hwang J, Kim D-H. Microphysiological blood–brain barrier systems for disease modeling and drug development. *Adv Healthc Mater* 2024;13:e2303180. 10.1002/adhm.202303180. 38430211 PMC11338747

[194] Zhang Y, Lu S-M, Zhuang J-J, Liang L-G. Advances in gut–brain organ chips. *Cell Prolif* 2024;57:e13724. 10.1111/cpr.13724. 39086147 PMC11503250

[195] Fakhri Bafghi MS, Khoshnam Rad N, Roostaei G, Nikfar S, Abdollahi M. The reality of modeling irritable bowel syndrome: progress and challenges. *Expert Opin Drug Discov* 2025;20:433–45. 10.1080/17460441.2025.2481264. 40162721

[196] Ahn K, Park H-S, Choi S, Lee H, Choi H, Hong SB, et al. Differentiating visceral sensory ganglion organoids from induced pluripotent stem cells. *Nat Methods* 2024;21:2135–46. 10.1038/s41592-024-02455-8. 39438735

[197] Kim NY, Lee HY, Choi YY, Mo SJ, Jeon S, Ha JH, et al. Effect of gut microbiota-derived metabolites and extracellular vesicles on neurodegenerative disease in a gut–brain axis chip. *Nano Converg* 2024;11:7. 10.1186/s40580-024-00413-w. 38340254 PMC10858859

[198] Lyu Z, Park J, Kim K-M, Jin H-J, Wu H, Rajadas J, et al. A neurovascular-unit-on-a-chip for the evaluation of the restorative potential of stem cell therapies for ischaemic stroke. *Nat Biomed Eng* 2021;5:847–63. 10.1038/s41551-021-00744-7. 34385693 PMC8524779

[199] Yan M, Man S, Sun B, Ma L, Guo L, Huang L, et al. Gut liver brain axis in diseases: the implications for therapeutic interventions. *Signal Transduct Target Ther* 2023;8:443. 10.1038/s41392-023-01673-4. 38057297 PMC10700720

[200] Li M, Duan W, Hao X, Li S, Wang C, Liang Y, et al. Effects of esketamine on electrophysiology and metabolic reprogramming in brain organoids: insights into antidepressant mechanisms. *Mol Psychiatry* 2025;30:6107–18. 10.1038/s41380-025-03198-4. 40885845

[201] Lee HR, Sung JH. Multiorgan-on-a-chip for realization of gut–skin axis. *Biotechnol Bioeng* 2022;119:2590–601. 10.1002/bit.28164. 35750599

[202] Ko B, Son J, In Won J, Kang BM, Choi CW, Kim R, et al. Gut microbe-skin axis on a chip for reproducing the inflammatory crosstalk. *Lab Chip* 2025;25:2609–19. 10.1039/d4lc01010h. 40042226

[203] Brandauer K, Schweinitzer S, Lorenz A, Krauß J, Schobesberger S, Frauenlob M, et al. Advances of dual-organ and multi-organ systems for gut, lung, skin and liver models in absorption and metabolism studies. *Lab Chip* 2025;25:1384–403. 10.1039/d4lc01011f. 39973270

[204] Wang J, Zhang H, Qu Y, Yang Y, Xu S, Ji Z, et al. An eighteen-organ microphysiological system coupling a vascular network and excretion system for drug discovery. *Microsyst Nanoeng* 2025;11:89. 10.1038/s41378-025-00933-3. 40368882 PMC12078732

[205] Sajin D, Moonshi SS, Cha H, Akther F, Zhang J, Nguyen N-T, et al. Multi-organ-on-a-chip: the gut and inflammatory diseases. *ACS Biomater Sci Eng* 2025;11:5330–42. 10.1021/acsbiomaterials.5c00208. 40799030

[206] Ziaka M, Exadaktylos A. Exploring the lung-gut direction of the gut-lung axis in patients with ARDS. *Crit Care Lond Engl* 2024;28:179. 10.1186/s13054-024-04966-4. PMC11131229 38802959

[207] Rae B, Bood V, Dijk H-J, Vasse GF, Melgert BN, Nagelkerke A, et al. Interorgan communication between lung and colorectal epithelial cells studied using a novel multi-organ-on-chip system. *Compr Physiol* 2025;15:e70051. 10.1002/cph4.70051. 40948361 PMC12434799

[208] Giordano L, Mihaila SM, Eslami Amirabadi H, Masereeuw R. Microphysiological systems to recapitulate the gut–kidney axis. *Trends Biotechnol* 2021;39:811–23. 10.1016/j.tibtech.2020.12.001. 33419585

[209] Mendes M, Morais AS, Carlos A, Sousa JJ, Pais AC, Mihăilă SM, et al. Organ-on-a-chip: quo vademus? Applications and regulatory status. *Colloids Surf B Biointerfaces* 2025;249:114507. 10.1016/j.colsurfb.2025.114507. 39826309

[210] Thorel L, Perréard M, Florent R, Divoux J, Coffy S, Vincent A, et al. Patient-derived tumor organoids: a new avenue for preclinical research and precision medicine in oncology. *Exp Mol Med* 2024;56:1531–51. 10.1038/s12276-024-01272-5. 38945959 PMC11297165

[211] Li J, Li Y, Song G, Wang H, Zhang Q, Wang M, et al. Revolutionizing cardiovascular research: human organoids as a beacon of hope for understanding and treating cardiovascular diseases. *Mater Today Bio* 2025;30:101396. 10.1016/j.mtbio.2024.101396. PMC11719415 39802826

[212] Probst C, Schneider S, Loskill P. High-throughput organ-on-a-chip systems: current status and remaining challenges. *Curr Opin Biomed Eng* 2018;6:33–41. 10.1016/j.cobme.2018.02.004.

[213] Schröter J, Deininger L, Lupse B, Richter P, Syrbe S, Mikut R, et al. A large and diverse brain organoid dataset of 1,400 cross-laboratory images of 64 trackable brain organoids. *Sci Data* 2024;11:514. 10.1038/s41597-024-03330-z. 38769371 PMC11106320

[214] Ravi K, Manoharan TJM, Wang K-C, Pockaj B, Nikkhah M. Engineered 3D ex vivo models to recapitulate the complex stromal and immune interactions within the tumor microenvironment. *Biomaterials* 2024;305:122428. 10.1016/j.biomaterials.2023.122428. 38147743 PMC11098715

[215] Man Y, Liu Y, Chen Q, Zhang Z, Li M, Xu L, et al. Organoids-on-a-chip for personalized precision medicine. *Adv Healthc Mater* 2024;13:e2401843. 10.1002/adhm.202401843. 39397335

[216] Tong M, Huang G, Zhuang S, Yu X, Hu D, Lin W, et al. Robotic micromanipulation for patterned and complex organoid biofabrication. *Sci Adv* 2025;11:eadz0808. 10.1126/sciadv.adz0808. 40911681 PMC12412664

[217] Diosdi A, Toth T, Harmati M, Istvan G, Schrettner B, Hapek N, et al. HCS-3DX, a next-generation AI-driven automated 3D-oid high-content screening system. *Nat Commun* 2025;16:8897. 10.1038/s41467-025-63955-5. 41057315 PMC12504613

[218] Hong Y, Li L, Yan L, Bai L, Su J, Zhang X. Parabiosis, assembloids, organoids (PAO). *Adv Sci Weinh Baden-Wurtt Ger* 2025;13:e11671. 10.1002/advs.202511671. PMC12866807 41014607

[219] Huang P, Lan H, Liu B, Mo Y, Gao Z, Ye H, et al. Transformative laboratory medicine enabled by microfluidic automation and artificial intelligence. *Biosens Bioelectron* 2025;271:117046. 10.1016/j.bios.2024.117046. 39671961

[220] Wang Y, Jeon H. 3D cell cultures toward quantitative high-throughput drug screening. *Trends Pharmacol Sci* 2022;43:569–81. 10.1016/j.tips.2022.03.014. 35504760

[221] Duarte LC, Figueredo F, Chagas CLS, Cortón E, Coltro WKT. A review of the recent achievements and future trends on 3D printed microfluidic devices for bioanalytical applications. *Anal Chim Acta* 2024;1299:342429. 10.1016/j.aca.2024.342429. 38499426

[222] Murata D, Arai K, Nakayama K. Scaffold-free bio-3D printing using spheroids as “bio-inks” for tissue (re-)construction and drug response tests. *Adv Healthc Mater* 2020;9:e1901831. 10.1002/adhm.201901831. 32378363

[223] Sharma C, Raza MA, Purohit SD, Pathak P, Gautam S, Corridon PR, et al. Cellulose-based 3D printing bio-inks for biomedical applications: a review. *Int J Biol Macromol* 2025;305:141174. 10.1016/j.ijbiomac.2025.141174. 39984091

[224] Fatimi A, Okoro OV, Podstawczyk D, Siminska-Stanny J, Shavandi A. Natural hydrogel-based bio-inks for 3D bioprinting in tissue engineering: a review. *Gels Basel Switz* 2022;8:179. 10.3390/gels8030179. PMC8948717 35323292

[225] Fukushima T, Togasaki K, Hamamoto J, Emoto K, Ebisudani T, Mitsuishi A, et al. An organoid library unveils subtype-specific IGF-1 dependency via a YAP-AP1 axis in human small cell lung cancer. *Nat Cancer* 2025;6:874–91. 10.1038/s43018-025-00945-y. 40307487

[226] Yue SSK, Tong Y, Siu HC, Ho SL, Law SYK, Tsui WY, et al. Divergent lineage trajectories and genetic landscapes in human gastric intestinal metaplasia organoids associated with early neoplastic progression. *Gut* 2025;74:522–38. 10.1136/gutjnl-2024-332594. 39572083

[227] Issing C, Menche C, Richter MR, Mosa MH, von der Grün J, Fleischmann M, et al. Head and neck tumor organoid biobank for modelling individual responses to radiation therapy according to the TP53/HPV status. *J Exp Clin Cancer Res CR* 2025;44:85. 10.1186/s13046-025-03345-3. 40045309 PMC11881459

[228] Dayton TL, Alcala N, Moonen L, den Hartigh L, Geurts V, Mangiante L, et al. Druggable growth dependencies and tumor evolution analysis in patient-derived organoids of neuroendocrine neoplasms from multiple body sites. *Cancer Cell* 2023;41:2083–2099.e9. 10.1016/j.ccell.2023.11.007. 38086335

[229] Beumer J, Geurts MH, Lamers MM, Puschhof J, Zhang J, van der Vaart J, et al. A CRISPR/Cas9 genetically engineered organoid biobank reveals essential host factors for coronaviruses. *Nat Commun* 2021;12:5498. 10.1038/s41467-021-25729-7. 34535662 PMC8448725

[230] Geurts MH, de Poel E, Amatngalim GD, Oka R, Meijers FM, Kruisselbrink E, et al. CRISPR-based adenine editors correct nonsense mutations in a cystic fibrosis organoid biobank. *Cell Stem Cell* 2020;26:503–510.e7. 10.1016/j.stem.2020.01.019. 32084388

[231] Tindle C, Fonseca AG, Taheri S, Katkar GD, Lee J, Maity P, et al. A living organoid biobank of patients with crohn’s disease reveals molecular subtypes for personalized therapeutics. *Cell Rep Med* 2024;5:101748. 10.1016/j.xcrm.2024.101748. 39332415 PMC11513829

[232] Nakanishi A, Toyama S, Onozato D, Watanabe C, Hashita T, Iwao T, et al. Effects of human induced pluripotent stem cell-derived intestinal organoids on colitis-model mice. *Regen Ther* 2022;21:351–61. 10.1016/j.reth.2022.08.004. 36161099 PMC9471335

[233] Shin YJ, Safina D, Zheng Y, Levenberg S. Microvascularization in 3D human engineered tissue and organoids. *Annu Rev Biomed Eng* 2025;27:473–98. 10.1146/annurev-bioeng-103023-115236. 40310885

[234] de Jongh D, Massey EK, VANGUARD consortium, Bunnik EM. Organoids: a systematic review of ethical issues. *Stem Cell Res Ther* 2022;13:337. 10.1186/s13287-022-02950-9. 35870991 PMC9308907

[235] Weidema J, de Vries M, Mummery C, de Graeff N. The ethical aspects of human organ-on-chip models: a mapping review. *Stem Cell Rep* 2025;20:102686. 10.1016/j.stemcr.2025.102686. PMC12790741 41173006

[236] Pașca SP, Arlotta P, Campbell P, Charo A, Evans JH, Farahany N, et al. The need for a global effort to attend to human neural organoid and assembloid research. *Science* 2025;390:574–7. 10.1126/science.aeb1510. 41196981

[237] Chen HI, Wolf JA, Blue R, Song MM, Moreno JD, Ming G-L, et al. Transplantation of human brain organoids: revisiting the science and ethics of brain chimeras. *Cell Stem Cell* 2019;25:462–72. 10.1016/j.stem.2019.09.002. 31585092 PMC7180006

[238] Ding L, Xiao Z, Gong X, Peng Y. Knowledge graphs of ethical concerns of cerebral organoids. *Cell Prolif* 2022;55:e13239. 10.1111/cpr.13239. 35582763 PMC9357362

[239] Fujimori K, Yamanaka S, Shimada K, Matsui K, Kawagoe S, Kuroda T, et al. Generation of human-pig chimeric renal organoids using iPSC technology. *Commun Biol* 2024;7:1278. 10.1038/s42003-024-06986-w. 39375428 PMC11458617

[240] van Daal M, de Kanter A-FJ, Bredenoord AL, de Graeff N. Personalized 3D printed scaffolds: the ethical aspects. *New Biotechnol* 2023;78:116–22. 10.1016/j.nbt.2023.10.006. 37848162

[241] Schäffers OJM, Gribnau J, van Rijn BB, Bunnik EM. Ethical considerations for advancing research using organoid models derived from the placenta. *Hum Reprod Update* 2025;31:392–401. 10.1093/humupd/dmaf007. 40096642 PMC12209499

